# Development and validation of a type 2 diabetes model to estimate the cost-effectiveness of diabetes interventions across the care continuum

**DOI:** 10.1017/S0266462325100172

**Published:** 2025-06-02

**Authors:** Megan Wiggins, Jeff Round, Erin Kirwin

**Affiliations:** 1 Institute of Health Economics, Edmonton, AB, Canada; 2 Faculty of Medicine and Dentistry, University of Alberta, Edmonton, AB, Canada; 3 Health Organisation, Policy, and Economics, School of Health Sciences, University of Manchester, Manchester, UK

**Keywords:** diabetes mellitus, type 2, computer simulation, preventive care, microsimulation, cost-effectiveness analysis

## Abstract

**Objectives:**

The aim of this study is to develop a patient-level model for type 2 diabetes mellitus (T2DM) progression that can estimate the cost-effectiveness of T2DM interventions from prevention to management.

**Methods:**

We developed an individual-level microsimulation model, the Institute of Health Economics Diabetes Model (IHE-DM), that simulates: (i) T2DM progression from normal glucose tolerance (NGT) to T2DM, (ii) the occurrence and timing of eight comorbidities and death, and (iii) the correlated progression of risk factors over time. We report model validation and use a case study to investigate the cost-effectiveness of a hypothetical T2DM prevention program.

**Results:**

The internal validation indicated excellent performance with mean absolute differences between the predicted and observed values for all endpoints of less than 1 percent. External validation results were mixed. The model under-predicted cumulative T2DM incidence in the first 8 years, predicted well from years eight through eleven, and over-predicted from years twelve through fifteen. Our case study estimated an incremental net monetary benefit of CAD 2,701 (USD 2,289) (95% Uncertainty Interval: CAD 1,316 to 4,000 [USD 1,115 to 3,390]) over the 15-year time horizon.

**Conclusions:**

Prominent T2DM models focus on patients with diagnosed T2DM whereas our model simulates progression from NGT to T2DM and incorporates important correlations in the progression of risk factors. These adaptations allow us to evaluate preventative interventions and better capture the long-term impacts, filling an important gap in the evidence base. Our model can be used to inform future funding decisions for T2DM interventions across the care continuum.

## Introduction

Diabetes is a chronic metabolic disorder and is associated with a wide array of complications. In 2023, 10 percent of Canadians were living with diabetes. Type 2 diabetes mellitus (T2DM) is the more prevalent form, accounting for more than 90 percent of cases (1). Complications from diabetes can have a significant impact on the healthcare system. The estimated direct healthcare system costs associated with diabetes in Canada in 2020 were CAD 3.8 billion (USD 3.2 billion) (2).

Given these substantial economic and health implications, there is an increased interest in interventions to reduce T2DM and the related economic burden, with a particular emphasis on prevention, as preventative interventions have demonstrated effectiveness in delaying or potentially preventing the onset of T2DM (3;4). Evidence evaluating the potential health and economic outcomes of T2DM interventions is essential for helping policymakers make informed funding decisions, especially when comparing interventions aiming at preventing versus managing T2DM. Simulation modeling is particularly well suited to this, as T2DM interventions typically incur high upfront costs while the benefits associated with reducing the burden of disease and lowering healthcare costs are realized gradually over years or even decades.

Several T2DM simulation models have been published to evaluate the long-term cost-effectiveness of various T2DM interventions (5–28). However, the existing models have limitations in their ability to evaluate preventive interventions, as they do not simulate either health states prior to the diagnosis of T2DM (5–11;28), or the correlated progression of T2DM risk factors over time and comorbidities before and after T2DM onset (12–27).

While the implications of not simulating health states prior to the diagnosis of T2DM on model suitability for the evaluation of preventative interventions are self-evident, the implications of not simulating the correlated progression of T2DM risk factors merit further explanation. Studies have shown that many different risk factors are important in predicting T2DM-related comorbidities and death (5). Multiple risk factors are also used to identify individuals at a high risk of developing T2DM who can benefit from preventive interventions (29). Interventions affect multiple risk factors simultaneously, and the relationships between risk factors affect the risk of other comorbidities, such as cardiovascular disease in patients before they develop T2DM (30;31). Until recently, there has been a paucity of published studies on the correlated progression of T2DM risk factors over time (32;33). Modelers have had to make simplifying assumptions, such as holding risk factors constant or estimating T2DM progression conditioned on a single risk factor, such as impaired glucose tolerance or BMI (13;14), which can limit the ability of models to accurately predict long-term health outcomes.

We sought to develop a T2DM simulation model, the Institute of Health Economics Diabetes Model (IHE-DM), that addresses these gaps. First, our model simulates progression from normal glucose tolerance (NGT) to pre-T2DM to T2DM, allowing for the evaluation of interventions at any point in the care pathway, including preventative interventions. Second, it incorporates the correlated progression of patient-level risk factors across this full continuum. Finally, our model simulates comorbidities both before and after the onset of T2DM. This comprehensive approach allows us to capture the full benefits of interventions, including their impact on health before the onset of T2DM, and provides a robust tool for assessing the long-term health and economic outcomes of different T2DM interventions applied across the care continuum.

This paper aims to detail the development and validation of the IHE-DM. We also demonstrate its capabilities by investigating the long-term cost-effectiveness of a hypothetical lifestyle intervention-based T2DM prevention program.

## Methods

### Model overview

The IHE-DM is an individual-level discrete-time microsimulation model that simulates: (i) progression from NGT to T2DM, (ii) the occurrence and timing of T2DM-related comorbidities and mortality, and (iii) time-varying changes in individual-level T2DM risk factors.

All patients move through two model levels during each 1-year model cycle. This continues until either a user-defined time horizon (e.g., 25 years) is reached or the patient dies. The top level of the model determines the patient’s T2DM health state, where they can be in one of two independent T2DM health states determined by their HbA1c: (i) NGT/pre-T2DM, with an HbA1c of less than 6.5 percent, or (ii) T2DM, with an HbA1c of 6.5 percent or higher (34).

**Figure 1.**
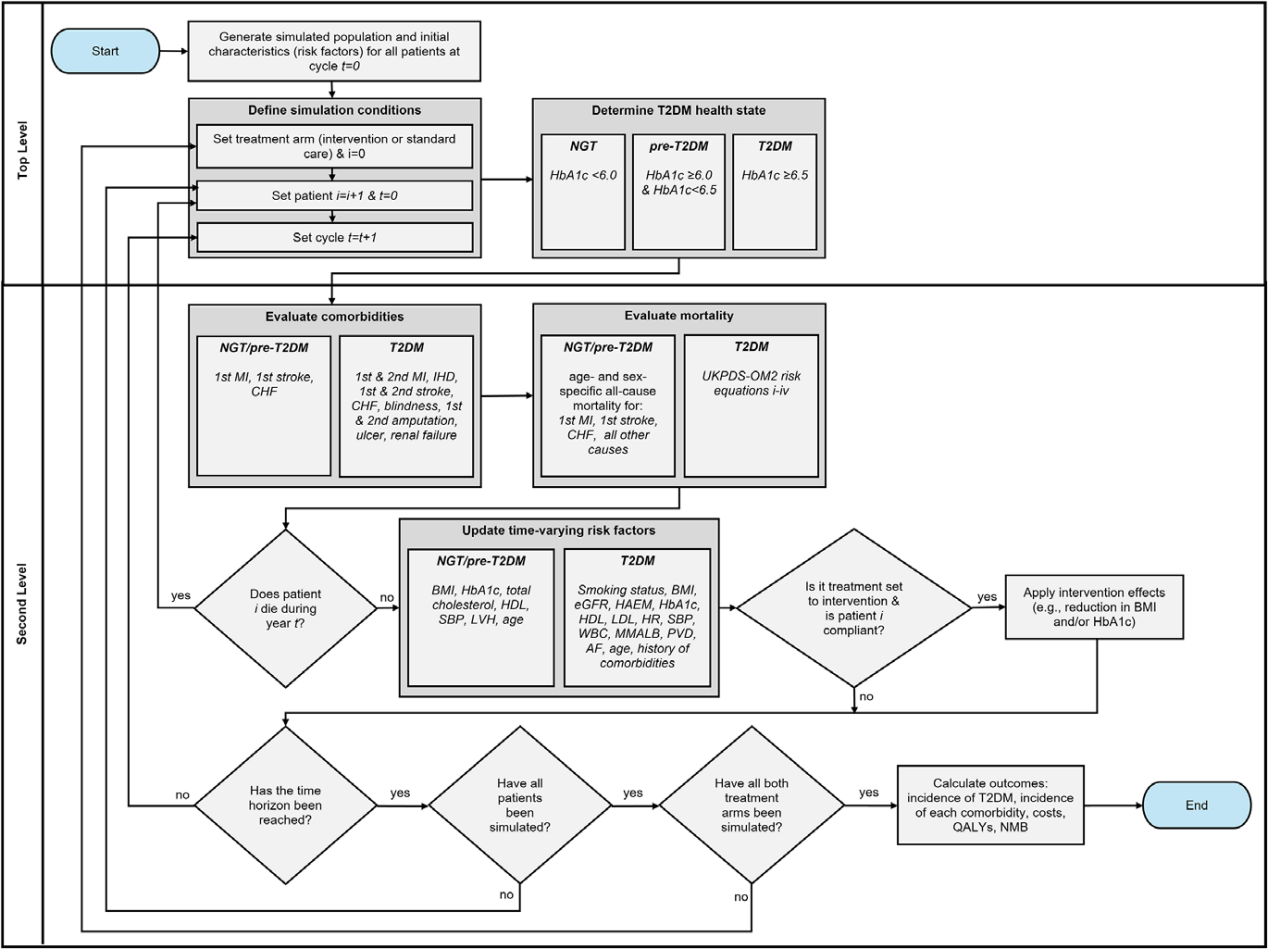
Model structure. AF, atrial fibrillation; BMI, body mass index; CHF, congestive heart failure; eGFR, estimated glomerular filtration rate; HAEM, hemoglobin; HbA1c, glycated hemoglobin; HDL, high-density lipoprotein; HR, heart rate; IHD, ischemic heart disease; LDL, low-density lipoprotein; LVH, left ventricular hypertrophy; MMALB, micro- or macro-albuminuria; MI, myocardial infarction; NGT, normal glucose tolerance; NMB, net monetary benefit; pre-T2DM, pre-type 2 diabetes mellitus; PVD, peripheral vascular disease; QALYs, quality-adjusted life-years; SBP, systolic blood pressure; T2DM, type 2 diabetes mellitus; UKPDS-OM2, United Kingdom Prospective Diabetes Study Outcomes Model Version 2; WBC, white blood cell count.

The second level of the model predicts the occurrence and timing of T2DM-related comorbidities (congestive heart failure [CHF], ischemic heart disease [IHD], blindness, renal failure, myocardial infarction [MI], stroke, amputation, diabetic ulcer) and death conditioned on the patient’s T2DM health state and their individual risk factors (e.g., age, history of comorbidities, BMI). Time-varying risk factors are also updated based on the patient’s T2DM health state and their individual risk factors in this section of the model. Figure 1 provides an overview of the model structure.

### Simulated population

The IHE-DM starts by generating initial characteristics for each patient by drawing random values from specific distributions corresponding to each characteristic (Section 1.2 of the Supplementary Material), which can be adjusted to simulate different populations. The patient’s initial HbA1c is used to determine their initial T2DM health state.

### Patients in the NGT/pre-T2DM state

**Figure 2.**
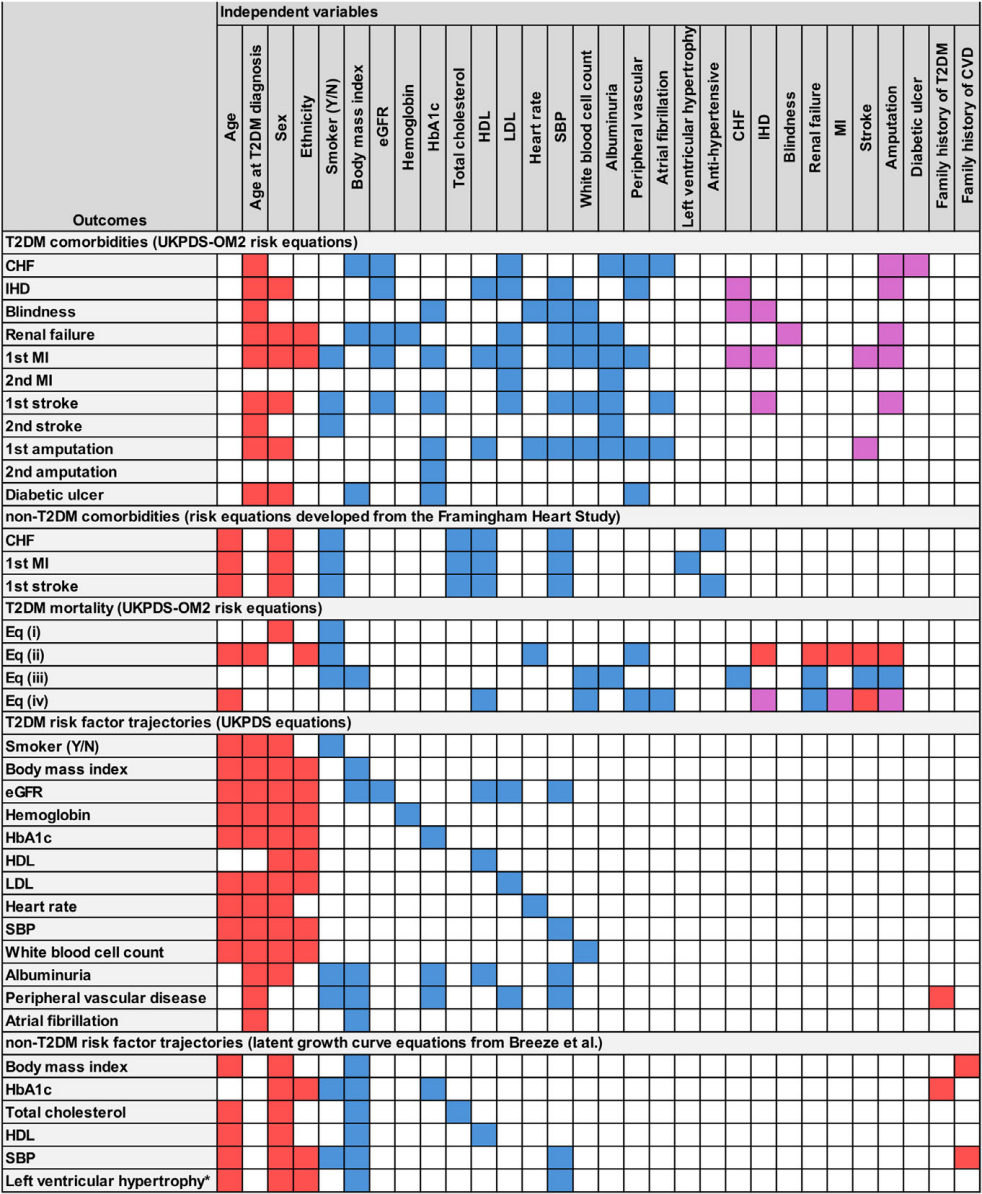
Independent variables are included in the model equations. Red shading indicates independent variables that are time-invariant (i.e., sex) or time logic (age)/current period, blue shading indicates independent variables that are calculated based on any prior period, purple shading indicates independent variables that are calculated based on the current period and any prior period; Albuminuria, micro- or macro-albuminuria; CHF, congestive heart failure; DBP, diastolic blood pressure; eGFR, estimated glomerular filtration rate; HbA1c, glycated hemoglobin; HDL, high-density lipoprotein; IHD, ischemic heart disease; LDL, low-density lipoprotein; LVH, left ventricular hypertrophy; MI, myocardial infarction; PVD, peripheral vascular disease; SBP, systolic blood pressure; T2DM, type 2 diabetes mellitus; WBC, white blood cell count. Mortality Eq (i): patients with no history of comorbidities and no comorbidities occur in the current year; Mortality Eq (ii): patients with no history of comorbidities but who experience one or more comorbidities in the current year; Mortality Eq (iii): patients with a history of comorbidities but experience no new comorbidities in the current year; Mortality Eq (iv): patients with a history of comorbidities who also experience at least one new comorbidity in the current year. *The prediction equation for LVH comes from an analysis by de Simone et al. (1994).

While in the NGT/pre-T2DM health state, patients can experience three comorbidities: MI (35), CHF, and stroke (36). The probability of each comorbidity occurring is calculated for each patient in every time cycle based on their individual characteristics (Figure 2). These probabilities are evaluated in a randomized order, allowing comorbidities to occur in a random sequence throughout the year. Once it occurs, CHF is considered a chronic comorbidity that persists through the remainder of the simulation or until death. Acute events include MI and stroke, which can occur once in the NGT/pre-T2DM health state and up to two times if the patient transitions to T2DM.

In any patient-year that a comorbidity occurs, the probability of death is based on the 1-year, age- and sex-specific all-cause mortality probability for that comorbidity (37–39). In patient-years without any comorbidities, the annual probability of death from all other causes is used, adjusted for mortality from MI, CHF, and stroke, which have already been accounted for (40).

#### Transitions to T2DM

At the end of each patient-year, the patient’s time-varying risk factors are updated based on their individual characteristics, using the appropriate equation (Figure 2). For patients with pre-T2DM, research indicates that the only significant difference in HbA1c progression between those who develop T2DM and those who do not occurs in the year before T2DM onset, with patients who transition to T2DM experiencing a sudden increase in HbA1c (41;42).

To incorporate these findings in the IHE-DM, if a patient’s HbA1c is between 6.0 and 6.4 percent (pre-T2DM), we include a 0.129 probability that their HbA1c will increase by 0.54 and they will transition to T2DM. Otherwise, their HbA1c is updated based on their individual characteristics using the HbA1c equation. The updated HbA1c is used at the start of the next model cycle to determine the patient’s T2DM health state (NGT/pre-T2DM or T2DM). A similar method of modeling T2DM state transitions has been used by Dall et al. (12).

### Patients in the T2DM state

For patients who transition to T2DM, the model uses the UKPDS-OM2 risk equations to predict comorbidities, mortality, and risk factor progression (5;33). These equations have been externally validated (43;44) and are widely used in T2DM simulation models (12;15). They were derived from data collected during the UKPDS trial and post-trial follow-up (45), which included newly diagnosed T2DM patients receiving treatments ranging from conventional glucose control through diet and lifestyle to intensive glucose control with metformin, sulfonylureas, insulin, or combination therapy – reflecting the current standard of care at the time of the study. Therefore, the IHE-DM assumes that all patients who progress to T2DM receive treatment consistent with the standard of care in the UKPDS.

Patients can experience eight comorbidities: CHF, IHD, blindness, renal failure, MI, stroke, amputation, and diabetic ulcer (5). The probability of each comorbidity occurring is calculated per patient-year based on the patient’s individual characteristics (Figure 2). These probabilities are evaluated in a randomized order, allowing comorbidities to occur in a random sequence throughout the year. Once they occur, chronic comorbidities (CHF, IHD, blindness, and renal failure), persist through the remainder of the simulation or until death. Acute events include MI, stroke, and amputation, each of which can occur up to two times.

The probability of mortality is calculated using one of four mutually exclusive equations for patients with: (i) no history of comorbidities and no comorbidities in the current year, (ii) no history of comorbidities but experience one or more comorbidities in the current year, (iii) history of comorbidities but no new comorbidities in the current year, and (iv) history of comorbidities and experience at least one new comorbidity in the current year.

At the end of each patient-year, time-varying risk factors are updated based on the patient’s individual characteristics (Figure 2).

### Costs and health-related quality of life

All costs are reported in Canadian dollars, inflated to 2022 prices using the healthcare component of the Canadian Consumer Price Index (46). The costs associated with T2DM and related comorbidities include the cost of inpatient hospital stays, outpatient visits, prescription drugs (both related to diabetes as well as other medications for patients aged 65 and older), emergency room visits, long-term care, and home care (7). To obtain the comorbidity costs of MI, CHF, and stroke for patients without T2DM, we use condition-specific cost ratios (47).

Baseline QALY values by age and sex and utility decrements for T2DM and comorbidities were sourced from literature as indicated in Table 1, which can be adjusted to reflect different populations.

**Table 1.** Model equations and parameters data sources

Component	NGT/pre-T2DM sources	T2DM sources
Comorbidities	*1st MI, CHF, and 1st stroke:* risk equations developed from the Framingham Heart Study (35;36)	*1st & 2nd MI, IHD, 1st & 2nd stroke, CHF, blindness, 1st & 2nd amputation, ulcer, renal failure:* UKPDS-OM2 risk equations (5)
Mortality	*Annual mortality probability following:* *1st MI* – age- and sex-specific probabilities from AMI Statistics (37) *CHF* – mean probability from McAlister et al. (39) *1st stroke* – age- and sex-specific probabilities from Vemmos et al. (38) *Age-sex-specific all other cause annual mortality probability:* Statistics Canada (40)	*All:* UKPDS-OM2 risk equations (5)
Risk factor progression	*Presence of LVH:* risk equation from de Simone et al. (48) *Age-sex-specific probability of receiving anti-hypertensive treatment:* Canadian Health Measures Survey (49) *All other (BMI, HbA1c, total cholesterol, HDL, SBP):* latent growth curve equations from Breeze et al. (31)	*Smoking status, BMI, eGFR, HAEM, HbA1c, HDL, LDL, HR, SBP, WBC, MMALB, PVD, AF:* UKPDS equations (33)
Transition to T2DM	*Based on HbA1c progression:* latent growth curve equation from Breeze et al. (31) and increases in HbA1c for those with pre-T2DM from Heianza et al. (41;42)	N/A
Costs	*Comorbidities (MI, CHF, and stroke) 1st year fatal, 1st year non-fatal, and subsequent years:* O’Reilly et al. (7) Choi et al. (47)	*T2DM with no comorbidities 1st year non-fatal and subsequent years:* O’Reilly et al. (7), Rosella et al. (50) *Diabetic foot ulcer 1st year non-fatal:* O’Brien et al. (51) *All other comorbidities 1st year fatal (MI & stroke only), 1st year non-fatal and subsequent years:* O’Reilly et al. (7)
QALYs	*Age-sex-specific baseline utility value:* 2018 Alberta Population Norms for EQ–5D–5L report (52) *Utility decrements for comorbidities (MI, CHF, and stroke):* Sullivan et al. (53)	*Age-sex specific baseline utility value:* 2018 Alberta Population Norms for EQ–5D–5L report (52) *Utility decrement for non-complicated T2DM:* Sullivan et al. (53) *Utility decrement for renal failure:* O’Reilly et al. (54) *Utility decrement for ulcer:* Sullivan et al. (53) *Utility decrements for all other comorbidities:* Alva et al. (55)

*Note*: AF, atrial fibrillation; BMI, body mass index; CHF, congestive heart failure; eGFR, estimated glomerular filtration rate; HAEM, hemoglobin; HbA1c, glycated hemoglobin; HDL, high-density lipoprotein; HR, heart rate; IHD, ischemic heart disease; LDL, low-density lipoprotein; LVH, left ventricular hypertrophy; MI, myocardial infarction; MMALB, micro- or macro-albuminuria; NGT, normal glucose tolerance; pre-T2DM, pre-type 2 diabetes mellitus; PVD, peripheral vascular disease; QALYs, quality-adjusted life-years; SBP, systolic blood pressure; T2DM, type 2 diabetes mellitus; UKPDS-OM2, United Kingdom Prospective Diabetes Study Outcomes Model Version 2; WBC, white blood cell count.

Each component of the model and the model parameters are described in greater detail in Section 1.3 of the Supplementary Material. Table 1 summarizes the parameters, equations, and data sources for each major component of the model. Figure 2 shows the individual-level characteristics that are predictors (independent variables) for comorbidities, mortality, and changes in risk factors over time. Probability values for model parameters not calculated using equations (e.g., probability of mortality in the NGT/pre-T2DM health state), along with cost and QALY values, are available in Section 1.3 of the Supplementary Material.

### Handling uncertainty

The IHE-DM deals with multiple levels of uncertainty. Initial characteristics and risk factors for each patient are generated by drawing random values from specified distributions to account for sampling uncertainty and patient heterogeneity (56). To minimize stochastic uncertainty, the model simulates a large number of individuals, which can be adjusted to ensure stable means for the outcomes of interest. After generating the initial population, parameter uncertainty in the clinical, cost, and QALY parameters is addressed through probabilistic sensitivity analysis (PSA), where 1,000 sets of input parameters are randomly sampled from the underlying probability distributions, and the results are simulated for each set of parameters. Further details on the PSA are provided in Section 1.5 of the Supplementary Material.

### Model validation

Through multiple iterations of model development, pressure testing, and extreme value analysis, we verified that all model elements were implemented correctly and that the model generated logical results (Section 1.4 of the Supplementary Material). Internal validation assessed how well the model predicted outcomes in the dataset used to derive the UKPDS-OM2 equations. External validation tested the model’s ability to predict T2DM occurrence in an external dataset from the Diabetes Prevention Program Outcome Study (DPPOS) (30;57).

#### Internal validation

Once patients transition to T2DM, the model uses the UKPDS-OM2 risk equations to predict T2DM-related comorbidities and death (5) and to simulate risk factor progression over time (33). To validate the implementation of these equations in the IHE-DM, we used the published baseline characteristics of UKPDS participants to generate a simulated cohort of 5,100 patients with newly diagnosed T2DM, reflecting the population size of the UKPDS. We then simulated risk factor progression, the development of comorbidities, and mortality for 20 years.

We compared the observed Kaplan–Meier (KM) cumulative failure probability from the UKPDS-OM2 to the simulated values from the model by plotting the simulated versus observed values for each of the comorbidities and mortality at years 5, 10, 15, and 20. If the model predicted the observed data perfectly for each endpoint, the plot points would fall along a 45-degree identity line. A majority of points falling above this line would indicate that the model overestimates the observed values. Conversely, a majority of points falling below the line would indicate that the model underestimates the observed values. We evaluated the accuracy of all predictions by fitting a regression line through the observed and predicted values for all endpoints, and estimating the intercept, slope coefficient, and the coefficient of determination (R^2^) (58). Perfectly accurate model predictions would have an intercept of zero, a slope coefficient of one, and an R^2^ of one. We used the same method to assess macrovascular (CHF, IHD, MI, stroke) and microvascular (blindness, renal failure, amputation, diabetic ulcer) endpoints separately. Last, we compared the simulated average risk factor progression for each risk factor with the average observed risk factor progression from the UKPDS over a 20-year follow-up period.

#### External validation

For external validation we required a longitudinal data source that reported: (i) a population without T2DM at baseline, (ii) baseline risk factors similar to those in our model, particularly HbA1c as this was the measure of blood glucose we used to determine T2DM health states, and (iii) outcomes consistent with our model outcomes (e.g., T2DM incidence). We selected the DPPOS (30;57), a long-term follow-up of the Diabetes Prevention Program trial (DPP), as it met these criteria.

We used the published baseline characteristics of the trial participants enrolled in the DDPOS to generate simulated cohorts of 5,100 patients for both the placebo and intensive lifestyle intervention (ILI) arm of the trial. When parameter values for initial risk factors were unavailable in the DPPOS, we used values from the UKPDS.

We simulated the annual effects of the ILI on BMI and HbA1c changes for the first 10 years following the intervention, as reported in the DPPOS 10-year follow-up (30). For patients with pre-T2DM, we applied a reduction to their probability of experiencing a sudden increase in HbA1c to more closely reflect the decrease in T2DM incidence observed in the ILI arm compared to the placebo arm in the DPPOS (Section 1.3 of the Supplementary Material).

We compared the mean simulated and observed KM cumulative incidence of T2DM annually for 15 years from randomization for each study arm (57). We calculated the average ratio of simulated to observed KM cumulative incidence. Values close to one would indicate good alignment between the model predictions and observed values from the DPPOS. Values greater than one would indicate that the model overpredicted the observed values, while values less than one would indicate underprediction.

### Case study: Cost-effectiveness of a potential T2DM prevention program

To illustrate the capabilities of the IHE-DM, we evaluated the 15-year cost-effectiveness of a hypothetical lifestyle intervention-based T2DM prevention program in Alberta, Canada. To align with the size of our validation cohorts, we simulated cohorts of 5,100 patients in each arm (standard care and intervention).

We based the T2DM prevention program on the ILI arm of the DPP trial. We simulated the annual effects of the ILI on BMI and HbA1c as described in the external validation section. The program compliance rate was assumed to be 50 percent (59;60). The program cost was assumed to be the same as the direct medical cost of the intensive lifestyle intervention in the DPP (61). We inflated the DPP cost to 2022 dollars using the US Consumer Price Index (62) and converted to Canadian dollars using the purchasing power parities (PPP) rate for 2022 (63).

Costs and QALYs were discounted at a 1.5 percent annual rate per the Canada Drug Agency Guidelines (64). We used a willingness-to-pay (WTP) threshold of CAD 30,000 (USD 25,424) (65). Section 1.5 of the Supplementary Material provides a complete description of the analysis.

## Results

### Internal validation

**Figure 3.**
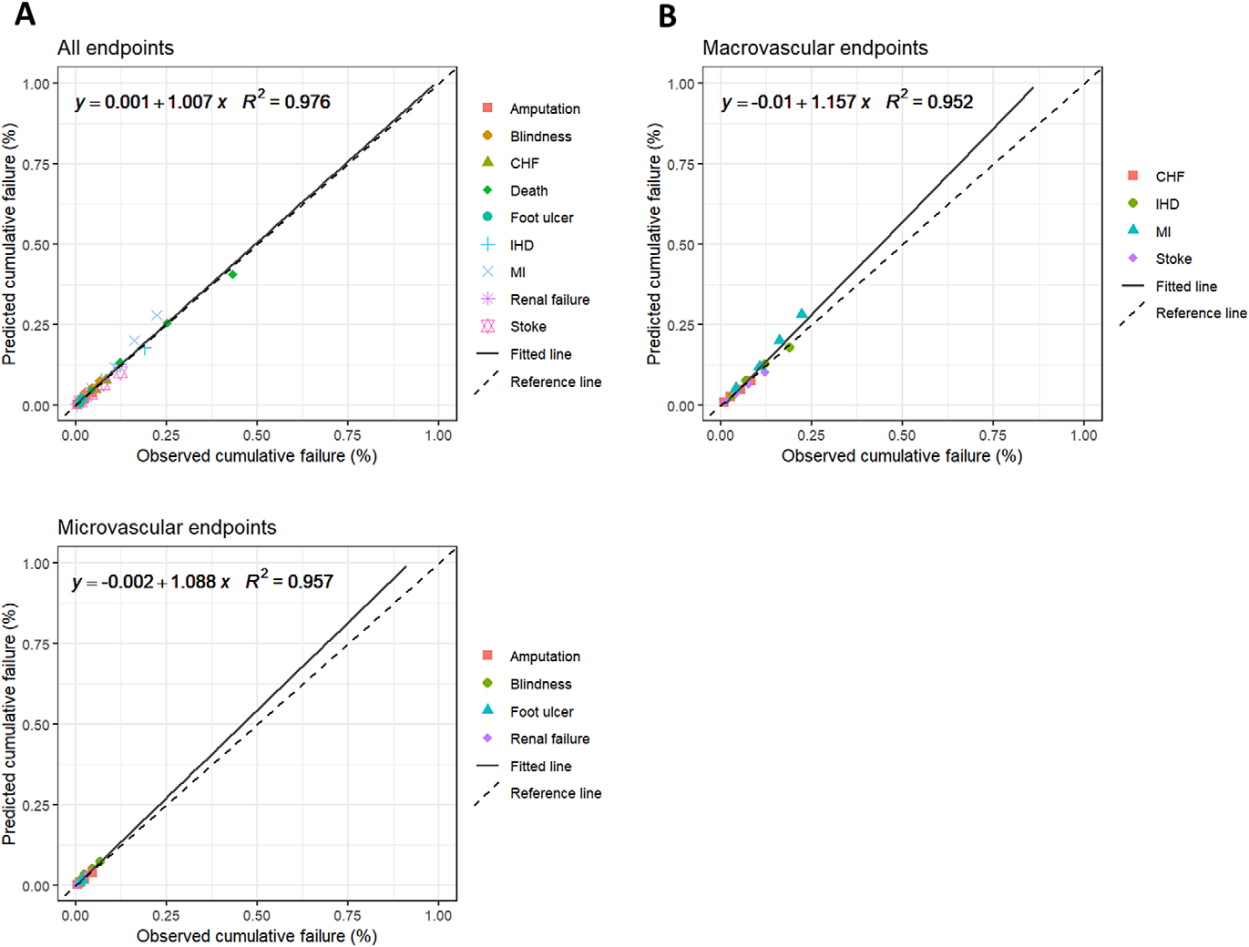
Predicted versus observed Kaplan–Meier cumulative failure probability, internal validation. Simulated mean Kaplan–Meier cumulative failure probability for each endpoint is calculated from 1,000 PSA iterations of 5,100 patients. The dashed line on the graphs represents the 45-degree reference line, and the solid line represents the fitted regression line. Each of the comorbidities and mortality are represented by three endpoints corresponding to years 5, 10, 15, and 20. CHF, congestive heart failure; IHD, ischemic heart disease; MI, myocardial infarction.

Results of the internal validation of the T2DM health state are shown in Figure 3, which plots the simulated and observed KM cumulative failure probability for each T2DM-related complication and mortality at years 5, 10, 15, and 20. Figure 3A plots all endpoints, Figure 3B plots microvascular endpoints only, and Figure 3C plots macrovascular endpoints only.

The intercepts are not statistically different from zero in all cases (all endpoints: 0.001 [95% confidence interval [CI]: −0.006, 0.008], microvascular endpoints: −0.002 [95% CI: −0.006, 0.003], and macrovascular endpoints: −0.010 [95% CI: −0.025, 0.006]). The slopes of the regression lines for each of the plots are not statistically different from one for both all endpoints and for microvascular endpoints (all endpoints: 1.007 [95% CI: 0.950, 1.065], microvascular endpoints: 1.088 [95% CI: 0.943, 1.233]). The slope for macrovascular endpoints is statistically significantly different from one (1.157 [95% CI: 1.008, 1.306]) but is relatively small in magnitude. The R^2^ value is greater than 0.95 in all cases.

The mean absolute difference between predicted and observed values was under 1 percent across all comorbidities and mortality. The exact simulated and observed values for each complication and mortality and the mean simulated and observed risk factor progression can be found in Section 2.1 of the Supplementary Material.

### External validation

**Figure 4.**
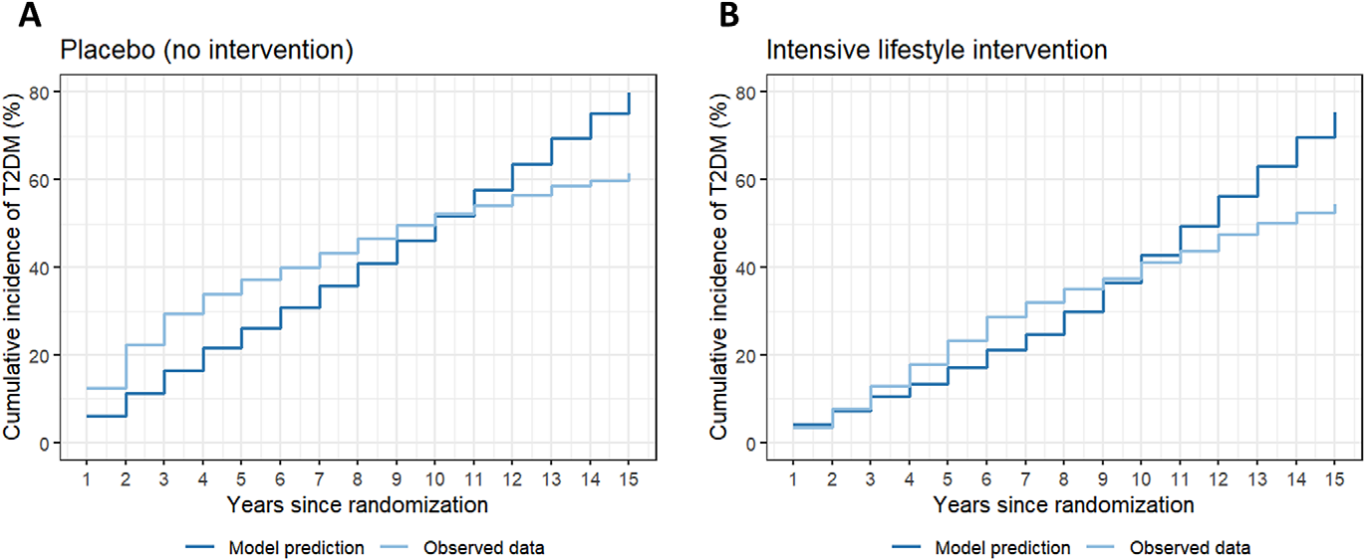
Simulated and observed cumulative incidence of T2DM, external validation. Simulated mean cumulative incidence of T2DM is calculated from 1,000 PSA iterations of 5,100 patients per iteration for each arm (placebo and intervention). PSA, probabilistic sensitivity analysis; T2DM, type 2 diabetes mellitus.

Figure 4 reports the mean simulated and observed KM cumulative incidence of T2DM annually for 15 years from randomization for the placebo arm (Figure 4A) and the ILI arm (Figure 4B). The ratio of simulated to observed KM cumulative incidence of T2DM is less than one in each year for the first 9 years in the ILI arm and in each year for the first 10 years in the placebo arm. At year 10, the simulated to observed KM cumulative incidence ratio is 1.04 and 0.99 for the ILI and placebo arms, respectively. From years 11 through 15, the simulated to observed KM cumulative incidence ratio is greater than one for both arms. At year 15, the simulated to observed KM cumulative incidence ratio is 1.38 and 1.30 for the ILI and placebo arms, respectively. The exact values for each year can be found in Section 2.1 of the Supplementary Material.

### Case study: Cost-effectiveness of a potential T2DM prevention program

We estimate that the hypothetical intervention has an incremental net monetary benefit of CAD 2,701 (USD 2,289) (95% Uncertainty Interval [UI]: CAD 1,316 to 4,000 [USD 1,115–3,390]) over the 15-year time horizon, becoming cost-effective in year 10. The intervention increases costs by CAD 541 (USD 458) (95% UI: CAD −123 to 1,230 [USD −145 to 1,042]) and increases QALYs by 0.11 (95% UI: 0.08–0.13). Additionally, the intervention reduces the T2DM event rate by 3,931 (95% UI: 2,946–5,016) cases per 100,000 person-years at risk compared to standard care over the 15-year model time horizon and reduces the event rates for all comorbidities. Detailed cost-effectiveness results and event rates are reported in Section 2.2 of the Supplementary Material alongside a completed Consolidated Health Economic Evaluation Reporting Standards 2022 (CHEERS 2022) checklist (66).

## Discussion

We developed a microsimulation model for T2DM to estimate the long-term health impacts, costs, and cost-effectiveness of T2DM interventions. Our model improves on other T2DM simulation models by: (i) simulating progression from NGT to pre-T2DM to T2DM, allowing for the evaluation of interventions at any point in the care pathway, (ii) simulating the correlated progression of multiple individual-level T2DM risk factors (e.g., BMI, HbA1c, blood pressure) over time to improve modelling of long-term health outcomes, and (iii) simulating comorbidities both before and after patients transition to T2DM to capture the full benefits of preventive interventions. Risk equations and parameter values were sourced from the literature. We illustrated the application of the model by evaluating the cost-effectiveness of a potential lifestyle intervention-based T2DM prevention program in Alberta, Canada.

It is difficult for policymakers to compare different interventions when their cost-effectiveness is evaluated using various models that may vary in terms of model states, assumptions, or input data. Multi-use disease models are increasingly recognized as important in addressing this challenge (67;68). In the case of T2DM, several such models have been developed, but they predominantly focus on patients with newly diagnosed T2DM (5–11;28). With the growing emphasis on preventive interventions, there is a need for multi-use T2DM models that can evaluate the potential health and economic outcomes of interventions across the full care continuum, from prevention to management. Our model fills this gap and can help policymakers make informed funding decisions, especially when comparing interventions aiming at preventing versus managing T2DM.

Internal and external validation of the model showed good model performance. In internal validation of the T2DM health state, the predicted KM cumulative failure probability of T2DM-related comorbidities and mortality aligned well with the observed KM cumulative failure probability of comorbidities and mortality in the UKPDS over 20 years. The mean absolute difference between predicted and observed values was under 1 percent across all comorbidities and mortality. The model predicted microvascular comorbidities slightly better than macrovascular, with mean absolute differences of 0.1 and 0.4 percent, respectively. Overall, our model demonstrates strong predictive performance for T2DM-related comorbidities and mortality within the UKPDS cohort.

For external validation, we compared the model predictions of the annual KM cumulative incidence of T2DM with the observed values in the 15-year follow-up study of the DPP trial. The model under-predicted T2DM incidence in the first 8 years, predicted reasonably well from years 8 through 11, and over-predicted from years 12 through 15 for both the placebo and the ILI arm. The over-prediction may stem from extrapolating findings from longitudinal studies. For patients with pre-T2DM, we applied an annual probability of 0.129 for a sudden increase in HbA1c, which results in progression to T2DM, based on longitudinal studies with mean follow-up durations of 5 and 9.5 years. Extrapolating these findings over a 15-year period may have led to the over-prediction of T2DM incidence over longer time periods. Additionally, the DPP diagnosed T2DM using fasting plasma glucose (FPG) and oral glucose tolerance test (OGTT), whereas our model used HbA1c to determine T2DM health states. While HbA1c is very specific, it is less sensitive than FPG and OGTT (69). Some evidence indicates that HbA1c may identify more people as having T2DM than FPG in Canada; however other studies suggest HbA1c may identify fewer people as having T2DM compared to FPG or OGTT (69), which could explain our model’s under-prediction of T2DM incidence from years 1 through 8. For these reasons, although the external validation indicates some discrepancies between the model predictions and the external dataset, interpretation of these discrepancies is difficult. External validation against other independent datasets that use HbA1c to diagnose T2DM would provide additional insights and will be an important future step in evaluating the model’s predictive performance. In addition, comparing our model’s performance with other published T2DM models through additional external validation activities, such as the Mount Hood Diabetes Challenge, will be an important area of future research (70).

Our study has limitations. First, we used the best available data in the literature to parameterize the IHE-DM. Nonetheless, the parameters and equations in the model are from multiple sources and are subject to the limitations of the source studies themselves. The characteristics of the populations in the source studies varied, potentially impacting the coherence of the various input parameters. Additionally, the primary source studies for predicting comorbidities, the FHS (1968–1987) and the UKPDS (1977–2002), are relatively dated. As standard care has improved over time (e.g., new medications and treatment devices for T2DM, statin use for high cholesterol), these equations may overestimate the risk for some comorbidities. Future work should endeavor to generate and validate newer values for model parameters and equations, ideally from the same population source for internal consistency.

Second, we used HbA1c to determine patients’ T2DM health state. Three tests can be used for T2DM diagnosis: HbA1c, FPG, and OGTT (34). We chose to use HbA1c because it is the measure of glucose control used in the UKPDS-OM2 equations (5), a primary source in our model. Using a different diagnostic test or a combination of tests may yield different results, either increasing or decreasing the occurrence of T2DM and related comorbidities. Consideration should be given to incorporating other measures of glucose control into future versions of the model.

Third, there can be a significant delay between the onset of T2DM and clinical diagnosis. Canadian data estimates that T2DM onset occurs 4–7 years before diagnosis (34). The IHE-DM does not take this into account. Once a patient’s HbA1c reaches 6.5 percent, they start the next model year with T2DM. From this point forward, it is assumed that the patient receives treatment for T2DM consistent with the treatment received by patients in the UKPDS. This likely results in an underestimate of the occurrence of comorbidities as patients are assumed to receive treatment for T2DM earlier than in practice.

The case study illustrates our model’s capabilities; however, a comprehensive cost-effectiveness analysis was beyond this paper’s scope. Nonetheless, it is important to acknowledge the case study’s limitations. We converted the direct medical cost of the ILI in the DPP to Canadian dollars using PPP. However, adapting US cost data to the Canadian setting is more complex than changing price weights, as US healthcare costs are generally higher than in Canada (64). We view the intervention cost in the case study as an upper bound for the cost of a similar program in Alberta. We estimated intervention compliance based on retention and participation in the US National DPP (59;60). However, retention rates can vary depending on implementation strategy (50). Future analysis of a lifestyle intervention-based T2DM prevention program should include sensitivity and scenario analysis to understand the impacts of the intervention cost and compliance on cost-effectiveness.

## Conclusions

This study presents the development and validation of a microsimulation model for T2DM. Whereas prominent T2DM models focus on newly diagnosed patients, our model contributes to the literature by simulating progression from normal glucose tolerance and/or pre-T2DM to T2DM. Our model also simulates the correlated progression of multiple T2DM risk factors over time and comorbidities before and after T2DM onset. These adaptations allow us to evaluate preventative interventions and more accurately capture the long-term impacts of interventions, filling an important gap in the evidence base. Our model can be used to inform future funding decisions and define target populations for T2DM interventions across the care continuum.

## Supporting information

Wiggins et al. supplementary material Wiggins et al. supplementary material

## Supplementary material

The supplementary material for this article can be found at http://doi.org/10.1017/S0266462325100172.

## References

[1] Diabetes Canada. Diabetes in Alberta. Ottawa (ON); 2023.

[2] Diabetes Canada. Diabetes in Canada. Ottawa (ON); 2020.

[3] Alouki K, Delisle H, Bermúdez-Tamayo C, Johri M. Lifestyle interventions to prevent type 2 diabetes: A systematic review of economic evaluation studies. J Diabetes Res. 2016;2159890. 26885527 10.1155/2016/2159890 PMC4738686

[4] Roberts S, Barry E, Craig D, et al. Preventing type 2 diabetes: Systematic review of studies of cost-effectiveness of lifestyle programmes and metformin, with and without screening, for pre-diabetes. BMJ Open. 2017;7:e017184. 10.1136/bmjopen-2017-017184 PMC5695352 29146638

[5] Hayes AJ, Leal J, Gray AM, Holman RR, Clarke PM. UKPDS outcomes model 2: A new version of a model to simulate lifetime health outcomes of patients with type 2 diabetes mellitus using data from the 30 year United Kingdom prospective diabetes study: UKPDS 82. Diabetologia. 2013;56:1925–1933. 23793713 10.1007/s00125-013-2940-y

[6] Willis M, Asseburg C, He J. Validation of economic and health outcomes simulation model of type 2 diabetes mellitus (ECHO-T2DM). J Med Econ. 2013;16:1007–1021. 23718682 10.3111/13696998.2013.809352

[7] O’Reilly D, Hopkins R, Blackhouse G, et al. Development of an Ontario diabetes economic model (ODEM) and application to a multidisciplinary primary care diabetes management program. Hamilton (ON): Onatio Ministry of Health and Long-term Care; 2006.

[8] The CDC diabetes cost-effectiveness group. Cost-effectiveness of intensive glycemic control, intensified hypertension control, and serum cholesterol level reduction for type 2 diabetes. JAMA. 2002;287:2542–2551. 12020335 10.1001/jama.287.19.2542

[9] Palmer AJ, Roze S, Valentine WJ, Minshall ME, Foos V, Lurati FM, et al. The CORE diabetes model: Projecting long-term clinical outcomes, costs and cost-effectiveness of interventions in diabetes mellitus (types 1 and 2) to support clinical and reimbursement decision-making. Curr Med Res Opin. 2004;Suppl 1:S5–26. 10.1185/030079904X1980 15324513

[10] Basu A, Sohn M-W, Bartle B, et al. Development and validation of the real-world progression in diabetes (RAPIDS) model. Med Decis Mak. 2019;39:137–151. 10.1177/0272989X18817521 PMC6624067 30654704

[11] Sobotka I, Cipriano L, Coyle D, Thiruchelvam D, Shah B. Population health value of being in target: Results from the Canadian multi-morbidity model for type 2 diabetes. Int J Popul Data Sci. 2024;9(5):2506.

[12] Dall TM, Storm MV, Semilla AP, et al. Value of lifestyle intervention to prevent diabetes and sequelae. Am J Prev Med. 2014;48:271–280. 25498548 10.1016/j.amepre.2014.10.003

[13] Galani C, Schneider H, Rutten FF. Modelling the lifetime costs and health effects of lifestyle intervention in the prevention and treatment of obesity in Switzerland. Int J Public Health. 2007;52:372–382. 18369000 10.1007/s00038-007-7014-9

[14] Gillies CL, Lambert PC, Abrams KR, et al. Different strategies for screening and prevention of type 2 diabetes in adults: Cost effectiveness analysis. BMJ. 2008;336:1180–1185. 18426840 10.1136/bmj.39545.585289.25 PMC2394709

[15] Lindgren P, Lindström J, Tuomilehto J, et al. Lifestyle intervention to prevent diabetes in men and women with impaired glucose tolerance is cost-effective. Int J Technol Assess Health Care. 2007;23:177–183. 17493303 10.1017/S0266462307070286

[16] Ackermann RT, Marrero DG, Hicks KA, et al. An evaluation of cost sharing to finance a diet and physical activity intervention to prevent diabetes. Diabetes Care. 2006;29:1237–1241. 16732002 10.2337/dc05-1709

[17] Caro JJ, Getsios D, Caro I, Klittich WS, O’Brien JA. Economic evaluation of therapeutic interventions to prevent type 2 diabetes in Canada. Diabet Med. 2004;21:1229–1236. 15498090 10.1111/j.1464-5491.2004.01330.x

[18] Hoerger TJ, Hicks KA, Sorensen SW, et al. Cost-effectiveness of screening for pre-diabetes among overweight and obese U.S. adults. Diabetes Care. 2007;30:2874–2879. 17698614 10.2337/dc07-0885

[19] Kaasalainen K, Kalmari J, Ruohonen T. Developing and testing a discrete event simulation model to evaluate budget impacts of diabetes prevention programs. J Biomed Inform. 2020;111:103577. 32992022 10.1016/j.jbi.2020.103577

[20] Mortaz S, Wessman C, Duncan R, Gray R, Badawi A. Impact of screening and early detection of impaired fasting glucose tolerance and type 2 diabetes in Canada: A Markov model simulation. Clinicoecon Outcomes Res. 2012;4:91–97. 22553425 10.2147/CEOR.S30547 PMC3340109

[21] Neumann A, Lindholm L, Norberg M, et al. The cost-effectiveness of interventions targeting lifestyle change for the prevention of diabetes in a Swedish primary care and community based prevention program. Eur J Health Econ. 2016;18:905–919. 27913943 10.1007/s10198-016-0851-9 PMC5533851

[22] Palmer AJ, Tucker DMD. Cost and clinical implications of diabetes prevention in an Australian setting: A long-term modeling analysis. Prim Care Diabetes. 2011;6:109–121. 22153888 10.1016/j.pcd.2011.10.006

[23] Palmer AJ, Roze S, Valentine WJ, et al. Intensive lifestyle changes or metformin in patients with impaired glucose tolerance: Modeling the long-term health economic implications of the diabetes prevention program in Australia, France, Germany, Switzerland, and the United Kingdom. Clin Ther. 2004;26:304–321. 15038953 10.1016/s0149-2918(04)90029-x

[24] Pierse T, O’Neill S, Dinneen SF, O’Neill C. A simulation study of the economic and health impact of a diabetes prevention programme in Ireland. Diabet Med. 2021;38:e14540. 33576077 10.1111/dme.14540

[25] Roberts S, Craig D, Adler A, McPherson K, Greenhalgh T. Economic evaluation of type 2 diabetes prevention programmes: Markov model of low-and high-intensity lifestyle programmes and metformin in participants with different categories of intermediate hyperglycaemia. BMC Med. 2018;16:1–12. 10.1186/s12916-017-0984-4 PMC5798197 29378576

[26] Smith KJ, Hsu HE, Roberts MS, et al. Cost-effectiveness analysis of efforts to reduce risk of type 2 diabetes and cardiovascular disease in southwestern Pennsylvania, 2005–2007. Prev Chronic Dis. 2010;7:A109. 20712936 PMC2938403

[27] Vandenberghe D. Simulating lifestyle and medical interventions to prevent type-2 diabetes: An economic evaluation for Belgium. Eur J Health Econ. 2021;23:237–248. 34390431 10.1007/s10198-021-01362-5

[28] Hoerger TJ, Hilscher R, Neuwahl S, et al. A new type 2 diabetes microsimulation model to estimate long-term health outcomes, costs, and cost-effectiveness. Value Health. 2023;26:1372–1380. 37236396 10.1016/j.jval.2023.05.013 PMC11017333

[29] Centers for Disease Control and Prevention (CDC). *Requirements for CDC recognition.* CDC. Available from: https://www.cdc.gov/diabetes/prevention/requirements-recognition.htm.

[30] Diabetes Prevention Program Research Group, Knowler WC, Fowler SE, et al. 10-year follow-up of diabetes incidence and weight loss in the diabetes prevention program outcomes study. Lancet. 2009;374:1677–1686. 19878986 10.1016/S0140-6736(09)61457-4 PMC3135022

[31] Breeze P, Squires H, Chilcott J, et al. A statistical model to describe longitudinal and correlated metabolic risk factors: The Whitehall II prospective study. J Public Health (Oxf). 2015;38:679–687. 10.1093/pubmed/fdv160 PMC6092879 28158533

[32] Palmer AJ. Computer Modeling of diabetes and its complications: A report on the fifth Mount Hood challenge meeting. Value Health. 2013;16:670–685. 23796302 10.1016/j.jval.2013.01.002

[33] Leal J, Alva ML, Gregory V, et al. Estimating risk factor progression equations for the UKPDS outcomes model 2 (UKPDS 90). Diabet Med. 2021;38:e14656. 34297424 10.1111/dme.14656

[34] Diabetes Canada. Diabetes Canada 2018 clinical practice guidelines for the prevention and management of diabetes in Canada. Diabetes Canada; 2018.

[35] Anderson KM, Odell PM, Wilson PW, Kannel WB. Cardiovascular disease risk profiles. Am Heart J. 1991;121:293–298. 1985385 10.1016/0002-8703(91)90861-b

[36] D’Agostino RB Sr, Vasan RS, Pencina MJ, et al. General cardiovascular risk profile for use in primary care: The Framingham heart study. Circulation. 2008;117:743–753. 18212285 10.1161/CIRCULATIONAHA.107.699579

[37] AMI Statistics. Based on county of residence, case fatality of AMI. Deaths within 365 days (%), entire Sweden, age interval: 20–85+. National Board of Health and Welfare, editors. Stockhom (Sweden); 2022.

[38] Vemmos KN, Bots M, Tsibouris P, et al. Prognosis of stroke in the south of Greece: 1 year mortality, functional outcome and its determinants: The Arcadia stroke registry. J Neurol Neurosurg Psychiatry. 2000;69:595–600. 11032610 10.1136/jnnp.69.5.595 PMC1763387

[39] McAlister FA, Bakal JA, Kaul P, et al. Changes in heart failure outcomes after a province-wide change in health service provision a natural experiment in Alberta, Canada. Circ Heart Fail. 2013;6:76–82. 23230308 10.1161/CIRCHEARTFAILURE.112.971119

[40] Statistics Canada, editor. Table 13-10-0392-01 deaths and age-specific mortality rates, by selected grouped causes. Ottawa (ON); 2022.

[41] Heianza Y, Arase Y, Fujihara K, et al. Longitudinal trajectories of HbA1c and fasting plasma glucose levels during the development of type 2 diabetes: The Toranomon hospital health management Center study 7 (TOPICS 7). Diabetes Care. 2012;35:1050–1052. 22456865 10.2337/dc11-1793 PMC3329827

[42] Heianza Y, Arase Y, Fujihara K, et al. Screening for pre-diabetes to predict future diabetes using various cut-off points for HbA(1c) and impaired fasting glucose: The Toranomon hospital health management Center study 4 (TOPICS 4). Diabet Med. 2012;29:279–285. 10.1111/j.1464-5491.2012.03686.x 22510023

[43] Pagano E, Konings SRA, Di Cuonzo D, et al. Prediction of mortality and major cardiovascular complications in type 2 diabetes: External validation of UK prospective diabetes study outcomes model version 2 in two European observational cohorts. Diabetes Obes Metab. 2021;23:1084–1091. 33377255 10.1111/dom.14311

[44] Keng MJ, Leal J, Mafham M, et al. Performance of the UK prospective diabetes study outcomes model 2 in a contemporary UK type 2 diabetes trial cohort. Value Health. 2022;25:435–442. 35227456 10.1016/j.jval.2021.09.005 PMC8881217

[45] Holman RR, Paul SK, Bethel MA, Matthews DR, Neil HA. 10-year follow-up of intensive glucose control in type 2 diabetes. N Engl J Med. 2008;35:1577–1589. 10.1056/NEJMoa0806470 18784090

[46] Statistics Canada. Consumer price index (CPI). Ottawa (ON); 2023.

[47] Choi J, Booth G, Jung HY, et al. Association of diabetes with frequency and cost of hospital admissions: A retrospective cohort study. CMAJ Open. 2021;9:E406–E412. 10.9778/cmajo.20190213 PMC8084549 33863799

[48] de Simone G, Devereux RB, Roman MJ, Alderman MH, Laragh JH. Relation of obesity and gender to left ventricular hypertrophy in normotensive and hypertensive adults. Hypertension. 1994;23:600–6. 8175168 10.1161/01.hyp.23.5.600

[49] DeGuire J, Clarke J, Rouleau K, Roy J, Bushnik T. Blood pressure and hypertension. Ottawa (ON): Statistics Canada; 2019. 30785635

[50] Rosella LC, Lebenbaum M, Fitzpatrick T, et al. Impact of diabetes on healthcare costs in a population-based cohort: A cost analysis. Diabet Med. 2016;33:395–403. 26201986 10.1111/dme.12858 PMC5014203

[51] O’Brien JA, Patrick AR, Caro JJ. Cost of managing complications resulting from type 2 diabetes mellitus in Canada. BMC Health Serv Res. 2003;3:7. 12659641 10.1186/1472-6963-3-7 PMC153533

[52] University of Alberta School of Public Health. Alberta PROMS and EQ-5D research support unit. Alberta population norms for EQ-5D-5L. Edmonton (AB): University of Alberta; 2018.

[53] Sullivan PW, Lawrence WF, Ghushchyan V. A national catalog of preference-based scores for chronic conditions in the United States. Med Care. 2005;736–749. 15970790 10.1097/01.mlr.0000172050.67085.4f

[54] O’Reilly DJ, Xie F, Pullenayegum E, et al. Estimation of the impact of diabetes-related complications on health utilities for patients with type 2 diabetes in Ontario, Canada. Qual Life Res. 2011;20:939–943. 21221816 10.1007/s11136-010-9828-9

[55] Alva M, Gray A, Mihaylova B, Clarke P. The effect of diabetes complications on health-related quality of life: The importance of longitudinal data to address patient heterogeneity. Health Econ. 2014;23:487–500. 23847044 10.1002/hec.2930

[56] Dakin HA, Leal J, Briggs A, et al. Accurately reflecting uncertainty when using patient-level simulation models to extrapolate clinical trial data. Med Decis Mak. 2020;40:460–473. 10.1177/0272989X20916442 PMC7323001 32431211

[57] Diabetes Prevention Program Research Group. Long-term effects of lifestyle intervention or metformin on diabetes development and microvascular complications over 15-year follow-up: The diabetes prevention program outcomes study. Lancet Diabetes Endocrinol. 2015;3:866–875. 26377054 10.1016/S2213-8587(15)00291-0 PMC4623946

[58] Steyerberg EW, Vickers AJ, Cook NR, et al. Assessing the performance of prediction models: A framework for traditional and novel measures. Epidemiology. 2010;21:128–138. 20010215 10.1097/EDE.0b013e3181c30fb2 PMC3575184

[59] Cannon MJ, Masalovich S, Ng BP, et al. Retention among participants in the National Diabetes Prevention Program Lifestyle Change Program, 2012–2017. Diabetes Care. 2020;43(9):2042. 32616617 10.2337/dc19-2366 PMC11000538

[60] Ritchie ND, Baucom KJW, Sauder KA. Current perspectives on the impact of the National Diabetes Prevention Program: Building on successes and overcoming challenges. Diabetes Metab Syndr Obes. 2020;13:2949–2957. 32903871 10.2147/DMSO.S218334 PMC7445538

[61] Hernan WH, Brandle M, Zhang P, et al. Costs associated with the primary prevention of type 2 diabetes mellitus in the diabetes prevention program. Diabetes Care. 2003;26:36–47. 12502656 10.2337/diacare.26.1.36 PMC1402339

[62] U.S. Bureau of Labor Statistics CPI inflation calculator. Available from: https://www.bls.gov/data/inflation_calculator.htm.

[63] Organisation for Economic Co-operation and Development (OECD) Purchasing power parities (PPP) (indicator). Available from: https://data.oecd.org/conversion/purchasing-power-parities-ppp.htm.

[64] Canad a’s Drug Agency (CDA). Guidelines for the economic evaluation of health technologies: Canada, 4th ed. Ottawa (ON): CDA; 2017.

[65] Ochalek JM, Lomas JRS, Claxton KP. Assessing health opportunity costs for the Canadian health care systems. 2018.

[66] Husereau D, Drummond M, Augustovski F, et al. Consolidated health economic evaluation reporting standards 2022 (CHEERS 2022) statement: Updated reporting guidance for health economic evaluations. BMC Med. 2022;20(23). 10.1186/s12916-021-02204-0 PMC8753858 35022047

[67] Scholte M, Ramaekers B, Danopoulos E, et al. Challenges in the assessment of a disease model in the NICE single technology appraisal of Tirzepatide for treating type 2 diabetes: An external assessment group perspective. PharmacoEconomics. 2024;42(8):829–832. 38717708 10.1007/s40273-024-01394-8 PMC11249712

[68] Wang J, Pouwels X, Ramaekers B, et al. A blueprint for multi-use disease modeling in health economics: Results from two expert-panel consultations. PharmacoEconomics. 2024;42(7):797–810. 38613660 10.1007/s40273-024-01376-w PMC11180025

[69] Punthakee Z, Goldenberg R, Katz P. Definition, classification and diagnosis of diabetes, prediabetes and metabolic syndrome. Can J Diabetes. 2018;42(Suppl 1):S10–s5. 29650080 10.1016/j.jcjd.2017.10.003

[70] Mount Hood Diabetes Challenge Network. Economics, simulation modelling & diabetes. 2022. Available from: https://www.mthooddiabeteschallenge.com/.

