# Supplementary material for: Development and validation of a type 2 diabetes model to estimate the cost-effectiveness of diabetes interventions across the care continuum
Source: Int J Technol Assess Health Care. 2025 Jun 2;41(1):e36. doi: 10.1017/S0266462325100172 (PMC12188100; doi:10.1017/S0266462325100172)
Supplement: Wiggins et al. supplementary material [file S0266462325100172sup001.pdf]

Supplemental Material

1. Methods

1.1 Model Structure

The model structure used for this model was determined based on the results of a rapid literature review. Type 2 diabetes mellitus (T2DM) is known to be associated with a number of individual characteristics, biometrics, health behaviours, and other chronic health conditions. T2DM affects multiple organ systems, resulting in many different types of complications. These complications share common risk factors and are linked in that one complication may affect the likelihood of others.[1] In order to best reflect these associations within the model, a microsimulation structure was chosen. Microsimulation models provide an efficient way of simulating interdependent complications that can take into account a number of individual-level factors.[2] The microsimulation structure allows each patient to be simulated with a unique set of attributes. Based on these individual-level factors, transition probabilities specific to each patient in each time period can then be calculated, taking into account the effects of aging, biomarker interactions, and the presence of disease to simulate natural disease progression. Our model's basic structure is described below. A detailed description of the parameters used in each section of the model can be found in Section 1.3.

The model can be thought of as having two levels. The top-level governs the T2DM health state of the patient, where a patient can be in one of two independent T2DM health states, determined by their glycated hemoglobin (HbA1c) level: (i) normal glucose tolerance (NGT)/pre-T2DM, or (ii) T2DM. The second level of the model governs comorbidities, mortality, and time-varying risk factor progression conditioned on a patient's T2DM health state. The second level of the model is based on an integrated system of equations used to predict the occurrence and timing of T2DM-related comorbidities (congestive heart failure [CHF], ischemic heart disease [IHD], blindness, renal failure, myocardial infarction [MI], stroke, amputation, and diabetic ulcer) as well as death based on each patient's individual characteristics such as age and sex, time-varying risk factors such as HbA1c, and the patient's history of comorbidities. Additional equations are used to update time-varying risk factors, taking into account the effects of aging, biomarker

interactions, and the presence of disease (particularly T2DM) to simulate natural disease progression.

A patient enters the model with a set of observable risk factors, including demographics, biometrics such as HbA1c, and the presence of various chronic diseases and/or history of adverse medical events. Starting at the top level of the model, the patient's initial HbA1c is used to determine their initial T2DM health state. The patients then progress through the second level of the model as follows:

1. The patient-specific probability of experiencing one or more comorbidities is calculated using the appropriate equations determined by their T2DM health state. Based on this probability, a random number is drawn from a uniform distribution to determine whether each comorbidity occurs for this patient.
2. The patient's specific probability of death is calculated according to the appropriate equation, as determined by their T2DM health state, history of comorbidities, and the onset of comorbidities experienced in the current year (if any). Based on this probability, a random number is drawn from a uniform distribution to determine whether the patient dies.
3. The patients-specific time-varying risk factors, such as HbA1c, are updated according to the appropriate equation as determined by their T2DM health state. The treatment effects for those in the intervention arm are applied directly to the patient’s HbA1c and body mass index (BMI) in the second level of the model.

Costs and quality-adjusted life years (QALYs) are then applied to each patient, and the patient returns to the top level of the model where their T2DM health state for the next cycle is determined using their updated HbA1c. This process is repeated annually for each patient through the model time horizon or until death.

The comorbidities included in the model were determined based on established associations with T2DM and available risk equations identified in the rapid literature review. The systems of equations used to calculate patient-level probabilities and simulate disease progression were chosen based on common data sources found in modelling studies in the rapid literature review. The equations from United Kingdom Prospective Diabetes Study Outcomes Model Version 2

(UKPDS-OM2) are widely used in diabetes simulation models around the world[3-6] and have been tested at the Mount Hood Challenge meetings,[7] however they are specific to patients that have been diagnosed with T2DM and cannot be applied to patients with NGT and pre-T2DM. As such, the UKPDS-OM2 equations are used only for patients who have transitioned to T2DM and a different set of equations is used for patients in the NGT and pre-T2DM health states. The equations used for patients in the NGT and pre-T2DM health states come from published clinical trials, observational studies and published government statistics. A key source is the Framingham Health State (FHS), which is a common data source used in many modelling studies.[4, 6, 8]

1.2 Simulated Population

We generated the initial risk factors for each patient by drawing random values from specific distributions corresponding to each characteristic. The parameter values for each distribution were sourced from the literature. In cases where data from the baseline examination of Diabetes Prevention Program trial (DPP) participants was available, we utilized the mean and standard deviation of those characteristics,[9] except for total cholesterol, high-density lipoprotein (HDL), and systolic blood pressure (SBP). For these factors, we applied equations from Breeze et al. (2016)[10] to calculate each value based on other simulated characteristics such as HbA1c and BMI. We also used a logistic regression equation from Breeze et al. (2016)[10] to generate the probability of each patient having a family history of T2DM. When parameter values for initial risk factors were unavailable in the DPP or Breeze et al. studies, we relied on the first available values from the UKPDS.[11] To ensure realistic values, we applied both the DPP inclusion/exclusion criteria,[12] as well as typical clinical ranges for biomarkers to truncate the distributions as necessary. Canadian-specific data was used to determine the proportion of the initial population that comprises current smokers, individuals on anti-hypertensive treatment, those with a history of first stroke and/or first MI, and individuals with a family history of cardiovascular disease (CVD) as described in the subsection below. Additionally, we relied on Alberta data from the 2021 census,[13, 14] along with the 2018 clinical practice guidelines for T2DM and Indigenous peoples,[15] to generate the ethnicity/Indigenous identity of the initial population as described in the subsection below. Table 1 displays the parameter values and distributions used to generate the initial risk factors for the simulated population.

TABLE 1: Characteristics of simulated population

Risk factor	Value	Distribution	Source and notes
**Individual characteristics**			
Current age in years, mean (SD)	50.6 (10.7)	Truncated-normal<br>Lower bound: 25<br>Upper bound: 85	Diabetes Prevention Program Research Group (2009)[9]<br>Diabetes Prevention Program (1999)[12]
Afro-Caribbean ethnicity, %	5.7	Sample with replacement	Statistics Canada (2023)[107]
Asian Indian ethnicity, %	18.7	Sample with replacement	Statistics Canada (2023)[107]
Indigenous identity (First Nations, Metis, Inuit), %	17.0	Sample with replacement	Government of Alberta (2023)[108]<br>Crowshoe et al. (2018)[15]
Caucasian ethnicity, %	58.6	Sample with replacement	Calculated assuming the rest of the population is Caucasian.
Female, %	67.7	Sample with replacement	Diabetes Prevention Program Research Group (2009)[9]
Current smoker, %	11.0	Sample with replacement	Government of Canada (2022)[16]
**Biomarkers**			
Body mass index – female (kg/m2), mean (SD)	34.9 (6.9)	Truncated-normal<br>Lower bound: 25<br>Upper bound: 45	Diabetes Prevention Program Research Group (2009)[9]<br>Intervention inclusion/exclusion criteria
Body mass index – male (kg/m2), mean (SD)	32.0 (5.7)	Truncated-normal<br>Lower bound: 25<br>Upper bound: 40	Diabetes Prevention Program Research Group (2009)[9]<br>Intervention inclusion/exclusion criteria
Estimated glomerular filtration rate (ml/min/1.73m2), mean (SD)	84.9 (18.1)	Truncated-normal<br>Lower bound: 60<br>Upper bound: 110	Leal et al. (2021)[11]<br>Nation Kidney Foundation Inc. (2023)[17]
Hemoglobin (g/dL), mean (SD)	14.7 (1.4)	Truncated-normal<br>Lower bound: 12<br>Upper bound: 18	Leal et al. (2021)[11]<br>Canadian Blood Services (2023)[18]
HbA1c (%), mean (SD)	5.91 (0.5)	Truncated-normal<br>Lower bound: 6.0	Leal et al. (2021)[11]<br>Intervention inclusion/exclusion criteria

Risk factor	Value	Distribution	Source and notes
		Upper bound: 6.49	
Total Cholesterol (mmol/l), mean (SD)	7.72 (0.52)	-	Calculated using equations from Breeze et al. (2016)[10]
High-density lipoprotein cholesterol (mmol/l), mean (SD)	1.34 (0.21)	-	Calculated using equations from Breeze et al. (2016)[10]
Triglycerides (mmol/l), mean (IQR)	1.59 (1.12, 2.27)	Log-normal	Diabetes Prevention Program Research Group (2009)[9]
Low-density lipoprotein cholesterol (mmol/l), mean (SD)	6.0 (0.6)	-	Calculated as LDL=total cholesterol – High-density lipoprotein cholesterol -0.2 x triglycerides[19]
Heart rate (beats per minute), mean (SD)	81.3 (14.8)	Truncated-normal<br>Lower bound: 40<br>Upper bound: 125	Leal et al. (2021)[11]<br>American Heart Association Inc. (2023)[20]
Systolic blood pressure (mm Hg), mean (SD)	130.2 (6.84)	-	Calculated using equations from Breeze et al. (2016)[10]
Diastolic blood pressure (mm Hg), mean (SD)	78.3 (9.3)	Truncated-normal<br>Lower bound: 50<br>Upper bound: 105	Diabetes Prevention Program Research Group (2009)[9]<br>Diabetes Prevention Program (1999)[12]<br>American Heart Association Inc. (2023)[21]
White blood cell count (1x10-6/ml), mean (SD)	6.9 (2.1)	Truncated-normal<br>Lower bound: 3<br>Upper bound: 12	Leal et al. (2021)[11]<br>Canadian Cancer Society (2023)[22]
Presence of micro- or macro-albuminuria, %	10.7	Sample with replacement	Leal et al. (2021)[11]
**Medical events, n (%)**			
Atrial fibrillation	1.1	Sample with replacement	Leal et al. (2021)[11]
Left ventricular hypertrophy, %	2.54	-	de Simone et al. (1994)[23]
Peripheral vascular disease	8.4	Sample with replacement	Leal et al. (2021)[11]
Anti-hypertensive treatment, %	*Male*<br>Age 25 to 39: 47.5	Sample with replacement	Statistics Canada (2019)[24]

Risk factor	Value	Distribution	Source and notes
	Age 40 to 59: 70.5<br>Age 60 to 69: 86.2<br>Age 70+: 91.1<br>*Female*<br>Age 25 to 39: 65.2<br>Age 40 to 59: 74.8<br>Age 60 to 69: 83.8<br>Age 70+: 86.4		
Family history of cardiovascular disease, %	41.32	Sample with replacement	Calculated based on 8.5% Canadian adults age 20 and over live with diagnosed heart disease[25] (see subsection below for details)
Family history of type 2 diabetes, %	59.6	Sample with replacement	Calculated using equations from Breeze et al. (2016)[10]
History of first amputation, %	0	-	Assumed to be 0
History of second amputation, %	0	-	Assumed to be 0
History of blindness, %	0	-	Assumed to be 0
History of congestive heart failure, %	0 (0)	-	Diabetes Prevention Program (1999)[12]
History of ischemic heart disease, %	0 (0)	-	Diabetes Prevention Program (1999)[12]
History of first stroke, %	1.3	Sample with replacement	Public Health Agency of Canada (2019)[26]
History of second stroke, %	0	-	Assumed to be 0
History of first myocardial infarction, %	2.1	Sample with replacement	Public Health Agency of Canada (2017)[27]
History of second myocardial infarction, %	0	-	Assumed to be 0
History of ulcer, %	0	-	Assumed to be 0
History of renal failure, %	0 (0)	-	Diabetes Prevention Program (1999)[12]

Note: HbA1c: glycated hemoglobin

Family history of CVD

The probability that a simulated patient has a family history of CVD is calculated based on data from the Public Health Agency of Canada, showing that approximately 8.5% of Canadian adults aged 20 and over live with diagnosed heart disease.[25] We assume that each simulated patient's parents and grandparents (six people total) have an independent 8.5% probability of having CVD. Therefore, the probability that none of them has CVD can be calculated as follows:

P(None have CVD) = (1 - 0.085) * (1 - 0.085) * (1 - 0.085) * (1 - 0.085) * (1 - 0.085) * (1 - 0.085) = 0.5868

Now, we can find the probability that at least one of them has CVD:

P(At least one has CVD) = 1 - P(None have CVD) = 1 - 0.5868 = 0.4132

Therefore, we assume that the probability of a simulated patient having a family history of CVD is 41.32%.

Ethnicity and Indigenous identity

We utilized Alberta data from the 2021 census,[13, 14] and referenced the 2018 clinical practice guidelines for T2DM and Indigenous peoples[15] to determine the simulated population's ethnicity/Indigenous identity distribution. First, we find the percentage of the overall Alberta population belonging to specific ethnic groups, namely Afro-Caribbean, Asian Indian, and Indigenous identity (First Nations, Métis, Inuit). As these are the only ethnicities explicitly considered in the comorbidity, mortality, and risk factor transition equations used in the model, we assumed the remaining population to be Caucasian. Next, we estimated the percentage of the intervention population we expected for each ethnicity. To do this, we used age-standardized diabetes prevalence rates among First Nations individuals, Métis people, and Inuit people in comparison to the general population.[15] This allowed us to calculate the increased likelihood of people with Indigenous identity being represented in the intervention program. For patients of Afro-Caribbean and Asian Indian ethnicity in the DPP program, we assumed their representation to be proportional to the percentage of these ethnic groups in the overall Alberta population. The participants in the intervention, apart from the specified ethnicities, were assumed to be

Caucasian. The distribution of ethnicity and Indigenous identity in the DPP patient population is detailed in Table 2.

TABLE 2: Ethnicity and Indigenous identity in the intervention patient population

Ethnicity and Indigenous identity	Percent of the Alberta population	T2DM prevalence multiplier	Percent of the intervention population
Afro-Caribbean	5.7%[107]	-	5.7%
Asian Indian	18.7%[107]	-	18.7%
First Nations	3.5%[108]	3.5[15]	12.3%
Metis	3.1%[108]	1.4[15]	4.3%
Inuit	0.1%[108]	1[15]	0.1%
Multiple responses to Indigenous identity	0.1%[108]	3.5*	0.4%
Caucasian	68.8%	-	58.6%
**Total**	**100.0%**	**-**	**100.0%**

*We assume multiple responses to Indigenous identity had the same increased prevalence of diabetes as First Nations individuals

None of the comorbidity or risk factor transition equations in the IHE-DM explicitly considered Indigenous peoples. As Indigenous peoples are at higher risk developing T2DM, we calculate the probability of comorbidities and mortality for Indigenous people using the ethnicity variable in the applicable equations with the highest probability of the event occurring. We use a similar approach for simulating risk factor progression among Indigenous peoples, implicitly assuming the highest possible risk of progression for this population. While we do not explicitly model a higher probability of developing T2DM, we use the ethnicity variable in HbA1c equation that ensures the fastest possible progression of HbA1c. As a result, Indigenous peoples will have the highest likelihood of transitioning to T2DM in the model.

1.3 Parameters

The model uses several different parameter types. Cost and health-related quality of life (HRQoL) parameters attach values to T2DM health states and comorbidities within the model. Transition parameters govern patient movement between T2DM health states in the top level of the model, as well as the occurrence of comorbidities and mortality and time-varying risk factor progression in the second level of the model. The transition parameters can be further classified into four types: (i) T2DM state transitions, (ii) comorbidity transitions, (iii) mortality transitions,

and (iv) risk factor transitions. Finally, intervention effectiveness parameters impact patient outcomes by modifying transitions in the model.

Cost

The following section describes the costs associated with T2DM health status and comorbidities. All costs are reported in Canadian dollars inflated to 2022 prices using the health care component of the Canadian Consumer Price Index.[28] A 1.5% discount rate is applied to all cost estimates, according to the Canada's Drug Agency guidelines (CDA) guidelines.[29]

Health state and comorbidity costs

We obtained the costs associated with T2DM-related comorbidities, except ulcer, from O'Reilly et al. (2006).[30] This study utilized the Ontario Diabetes Database to identify patients diagnosed with T2DM between 1992 and 2002. The researchers followed these patients for a period of 10 years and used a linked administrative dataset to obtain hospital inpatient costs and non-inpatient costs, including outpatient visits, prescription drugs (both drugs related to diabetes and other medications for patients aged 65 and older), emergency room visits, long-term care, and home care. The costs specific to each comorbidity were divided into two time periods: (i) costs incurred within the first year of the comorbidity, further categorized into fatal and non-fatal occurrences for MI and stroke, and (ii) costs in subsequent years related to the long-term management of the comorbidity, encompassing subsequent events of the same type. As O'Reilly et al. (2006)[30] did not provide costs for diabetic ulcers, we used the estimated event costs for episodic diabetic foot ulcer from O'Brien et al. (2003).[31] The mean annual cost of T2DM-related complications is reported in Table 3.

TABLE 3: Comorbidity costs

Comorbidity	1st year fatal	1st year non-fatal	Subsequent years	Source
**NGT and pre-T2DM health states**				
Myocardial infarction	$11,179	$21,315	$3,333	O'Reilly et al. (2006)[30] Choi et al. (2021)[32]
Congestive heart failure	-	$19,499	$5,467	
Stroke	$9,016	$24,885	$3,453	
**T2DM health state**				
No comorbidities	-	$1,459	$1,459	O'Reilly et al. (2006)[30] Rosella et al. (2016)[33]
Myocardial infarction	$11,402	$21,741	$3,400	O'Reilly et al. (2006)[30]

Congestive heart failure	-	$19,889	$5,576	
Stroke	$10,729	$29,614	$4,109	
Ischemic heart disease	-	$6,805	$3,929	
Amputation	-	$45,938	$6,292	
Blindness	-	$3,638	$2,592	
Renal failure	-	$29,475	$13,378	
Ulcer	-	$3,143	-	O'Brien et al. (2003)[31]

Note: NGT: normal glucose tolerance; pre-T2DM: pre-type 2 diabetes mellitus; T2DM: type 2 diabetes mellitus

To obtain the comorbidity costs of MI, CHF, and stroke for patients without T2DM, we used cost ratios from Choi et al. (2021).[32] This study estimated the cost of hospital admission for the most common and costly conditions leading to hospital admission, comparing patients with diabetes to those without diabetes. The cost ratios (cost for patients with diabetes/costs for patients without diabetes) for these conditions were reported. To determine the comorbidity costs for MI and stroke in patients without T2DM, we applied these cost ratios to the costs associated with the respective T2DM-related comorbidity data from O'Reilly et al. (2006).[30] Since Choi et al. (2021)[32] did not provide a specific cost ratio for MI, we assumed the same cost ratio as that of CHF. Additionally, we assumed that these cost ratios would remain consistent for non-hospital admission-related costs, including the cost of long-term management associated with these comorbidities. We also assumed that the cost ratios were the same for patients with pre-T2DM and patients with NGT. The mean annual costs of MI, CHF, and stroke for patients without T2DM are reported in Table 3.

O'Reilly et al. (2006)[30] also reported that patients with T2DM who do not experience any T2DM-related comorbidities have an annual average healthcare cost of $2,363. To determine the incremental cost for patients with T2DM who do not experience any T2DM-related comorbidities compared to patients without T2DM who do not experience comorbidities, we referred to the cost data from Rosella et al. (2016).[33] This study identified incident diabetes cases from the Ontario Diabetes Database between 2004 and 2012 and matched them to control subjects without diabetes. Over an eight-year follow-up period, the direct healthcare costs for both the diabetes cases and non-diabetic controls were calculated. Using these cost estimates, we calculated the cost ratio (cost for diabetes cases/costs for controls). We applied this cost ratio to the annual average healthcare cost for patients with T2DM of $2,363 to obtain the average

annual healthcare cost for patients without T2DM. We then calculated an incremental cost of $1,459 for patients with T2DM who do not experience any T2DM-related comorbidities compared to patients without T2DM who do not experience comorbidities.

A 1.5% discount rate is applied to all cost estimates, according to CDA guidelines.[29]

Health-Related Quality of Life

The presence of chronic diseases such as T2DM and other comorbidities can reduce HRQoL. This model uses QALYs to capture these effects.

Baseline HRQoL values stratified by age and sex were from the 2018 Alberta Population Norms for EQ-5D-5L report.[34] The data sources for this report included six independent cross-sectional population-based surveys from the Health Quality Council of Alberta and Alberta Health. The Health Quality Council of Alberta data is from the Satisfaction and Experience with Healthcare Services survey from 2012, 2014, and 2016. The Alberta Health data is from the Alberta Community Health Survey from 2014, 2015, and 2016. The average EQ-5D-5L index scores by age and sex are given in Table 4.

TABLE 4: EQ-5D-5L mean index scores

Age group	Female	Male
25-44	0.87	0.88
45-64	0.82	0.83
65-74	0.82	0.83
75+	0.79	0.81

Source: Alberta PROMS and EQ-5D Research Support Unit[34]

Utility effects of multiple concurrent comorbidities could be incorporated in the model different ways (e.g., additive method, multiplicative method). The IHE-DM used an additive method consistent with the recommendations in the Mount Hood Diabetes Challenge Network reference simulation[7] and therefore used utility decrements for T2DM and comorbidities.

The utility decrements associated with transitioning to the T2DM health state were sourced from Sullivan et al. (2005) and O'Reilly et al. (2011). Sullivan created a national catalogue of preference-based scores for chronic conditions in the United States and estimated the utility decrement associated with T2DM without complications as 0.035.[35]

The utility decrements associated with comorbidities in the T2DM health state were sourced from the UKPDS, where seven rounds of EQ-5D questionnaires were administered between 1997 and 2007.[36] The UKPDS also tested whether the effects of complications within the first year of an event differed from the longer-term effects. The researchers found this was the case for MI but not for any other complications. Therefore, the utility decrement for MI was separated into short-term (within the first year of an event) and long-term decrements, whereas all other complications can be interpreted as permanent decrements. The UKPDS did not include estimates for renal failure and diabetic ulcer. The utility decrement for renal failure was sourced from O'Reilly et al. (2011), and the utility decrement for diabetic ulcer was sourced from Sullivan et al. (2005), which reported a utility decrement for chronic skin ulcer.[35, 37]

QALY estimates for patients in the T2DM health state were calculated by taking the mean index score for their respective age and sex from Table 4, subtracting the utility decrement for T2DM, and the utility decrements for each comorbidity they experience from Table 5. For example, the QALY estimate for a 60-year-old female who has T2DM, has CHF, and experiences an amputation in the current year is calculated as follows:

$$\text{QALY} = 0.82 - 0.035 - 0.101 - 0.172 = 0.512$$

TABLE 5: Utility decrements

Comorbidity	Utility decrement	Source
T2DM without complications	0.0350	Sullivan et al. (2005)[35]
**T2DM**		
Myocardial infarction (short-term)	0.0650	Alva et al. (2014)[36]
Myocardial infarction (long-term)	0.0000	
Congestive heart failure	0.1010	
Ischemic heart disease	0.0280	
Stroke	0.1650	
Blindness	0.0000	
Amputation	0.1720	
Renal failure	0.1018	O'Reilly et al. (2011)[37]
Ulcer	0.0317	Sullivan et al. (2005)[35]
**NGT and pre-T2DM**		
Myocardial infarction	0.0345	Sullivan et al. (2005)[35]
Congestive heart failure	0.0546	
Stroke	0.0483	

Note: NGT: normal glucose tolerance; pre-T2DM: pre-type 2 diabetes mellitus; T2DM: type 2 diabetes mellitus

Utility decrements for comorbidities in the NGT and pre-T2DM health states were from Sullivan et al. (2005).[35] QALY estimates for individuals in the NGT and pre-T2DM health states were calculated by taking the mean index score for their respective age and sex from Table 4 and subtracting the utility decrement for each comorbidity they experience from Table 5.

A 1.5% discount rate is applied to all QALY estimates, according to CDA guidelines.[29]

T2DM State Transitions

Transitions between T2DM health states in the top level of the model are determined by a patient's HbA1c level and the Alberta Health diagnostic criteria[38] as outlined in Table 6.

TABLE 6: T2DM diagnostic criteria

T2DM health state	HbA1c (%)
NGT	<6.0
Pre-T2DM	≥6.0 & <6.5
T2DM	≥6.5

Notes: HbA1c: glycated hemoglobin; NGT: normal glucose tolerance; pre-T2DM: pre-type 2 diabetes mellitus; T2DM: type 2 diabetes mellitus. Source: Diabetes Canada Clinical Practice Guidelines Expert Committee et al.[38]

Other diagnostic tests, such as Fasting Plasma Glucose (FPG) and Oral Glucose Tolerance Test (OGTT), can also be used to diagnose pre-T2DM and T2DM. The UKPDS-OM2 model was the primary source for prediction equations used to model T2DM progression once a patient is in the T2DM health state, and the UKPDS-OM2 equations use HbA1c. Thus, HbA1c was used to define the T2DM state in the model.

T2DM state transitions are structured to reflect research findings from Heianza et al. (2012a, 2012b). An observational study by Heianza et al. (2012a) examined ten years of longitudinal data and found that the annual rate of change in HbA1c among patients with NGT and/or pre-T2DM who do or do not develop T2DM only differs significantly in the year before T2DM onset. In the year before T2DM onset, sudden increases in HbA1c of 0.64 (SE 0.06) occurred in patients who transitioned to T2DM. For these patients, the average HbA1c in the year prior to diagnosis was 5.9%.[39] A separate study by Heianza et al. (2012b) found that the annual incidence rate of T2DM among patients with HbA1c levels of 6.0 to 6.4 was 12.9%.[40] Therefore, if a simulated patient's

HbA1c level was in the 6.0 to 6.4 range, that patient had a 12.92% probability of a 0.54 increase in HbA1c as opposed to the change in HbA1c calculated using the risk factor transition equation. If a patient's HbA1c level was not in the 6.0 to 6.4 range, their HbA1c level was calculated using the risk factor transition equation for their T2DM health state (see Figure 1). A similar method of modelling T2DM state transitions is used by Dall et al. (2014).[4]

Figure 1: Logical flow for updating HbA1c in the NGT/pre-T2DM health state

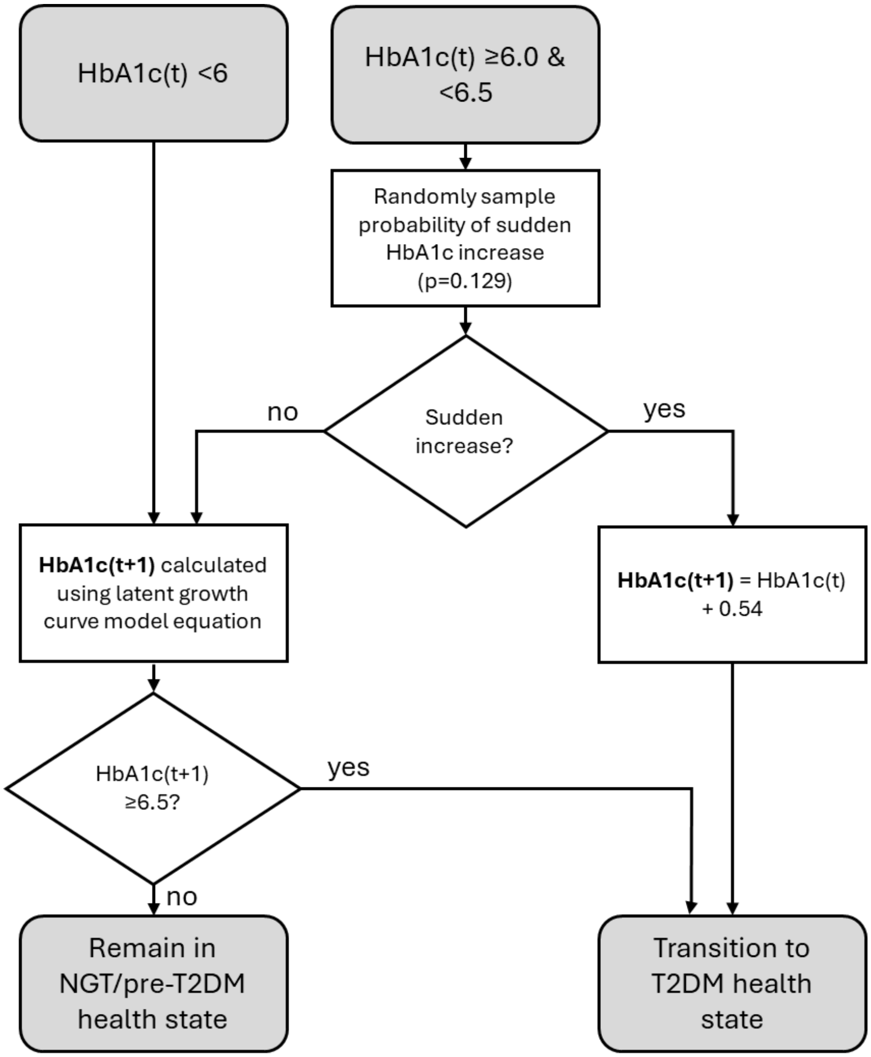

Notes: HbA1c(t): glycate hemoglobin value at the start of the current model cycle *t*; HbA1c(t+1): glycate hemoglobin value at the start of the next model cycle *t+1*.

Once patients transition to T2DM, they cannot transition back to the pre-T2DM or NGT health state regardless of their HbA1c value; that is, the model does not allow remission. This assumption is consistent with the literature review results, where 85% of the models reviewed did not allow "remission" based on the underlying knowledge that while remission is possible, it

rarely occurs.[41] The literature review results were less conclusive regarding allowing the transition from pre-T2DM back to NGT, with 50% of the models reviewed allowing this transition. The model allows patients to transition from pre-T2DM to NGT if their HbA1c level returns below the 6.0% threshold. This transition does not impact the comorbidity, mortality, or risk factor transition equations.

Comorbidity Transitions

The following section describes the comorbidity transition equations used in the second level of the model conditioned on a patient's T2DM health states as determined in the top level of the model. Each comorbidity has its own transition equation that takes into account various combinations of individual characteristics, biomarkers and history of comorbidities as independent variables, as outlined in Table 7.

TABLE 7: Variables in comorbidity transition equations

	T2DM comorbidity											non-T2DM adverse events/complications		
**Variable**	**CHF**	**IHD**	**Blind**	**Renal failure**	**1st MI**	**2nd MI**	**1st stroke**	**2nd stroke**	**1st LEA**	**2nd LEA**	**Ulcer**	**CHF**	**1st MI**	**1st stroke**
**Age**												t	t	t
**Age at T2DM diagnosis**	t	t	t	t	t		t	t	t		t			
**Sex**		t		t	t		t		t		t	t	t	t
**Ethnicity**				t	t									
**Smoke**					t-1		t-1	t-1				t-1	t-1	t-1
**BMI**	t-1			t-1							t-1			
**eGFR**	t-1	t-1		t-1	t-1		t-1							
**HAEM**				t-1										
**HbA1c**			t-1		t-1		t-1		t-1	t-1	t-1			
**TCHOL**												t-1	t-1	t-1
**HDL**		t-1			t-1				t-1			t-1	t-1	t-1
**LDL**	t-1	t-1		t-1	t-1	t-1	t-1							
**HR**			t-1						t-1					
**SBP**		t-1	t-1	t-1	t-1		t-1		t-1			t-1	t-1	t-1
**WBC**			t-1	t-1	t-1		t-1		t-1					

**MMALB**	t-1			t-1	t-1	t-1	t-1	t-1	t-1					
**PVD**	t-1	t-1			t-1				t-1		t-1			
**AF**	t-1						t-1		t-1					
**LVH**													t-1	
**HYP-TRT**												t-1		t-1
**CHF**		t, t-1	t, t-1		t, t-1									
**IHD**			t, t-1		t, t-1		t, t-1							
**Blind**				t, t-1										
**Stroke**					t, t-1				t, t-1					
**LEA**	t, t-1	t, t-1		t, t-1	t, t-1		t, t-1							
**Ulcer**	t, t-1													

Notes: 't' indicates time-invariant (i.e., sex) or time logic (age)/current period, 't-1' indicates any prior period; AF: atrial fibrillation; Blind: blindness; BMI: body mass index; CHF: congestive heart failure; DBP: diastolic blood pressure; eGFR: estimated glomerular filtration rate; HAEM: hemoglobin; HbA1c: glycate hemoglobin; HDL: high-density lipoprotein; HR: heart rate; HYP-TRT: anti-hypertensive treatment; IHD: ischemic heart disease; LEA: lower extremity amputation; LDL: low-density lipoprotein; LVH: left ventricular hypertrophy; MI: myocardial infarction; MMALB: micro- or macro-albuminuria; PVD: peripheral vascular disease; SBP: systolic blood pressure; TCHOL: total cholesterol; T2DM: type 2 diabetes mellitus; WBC: white blood cell count. *Sources:* Hayes et al.,[3] Anderson et al.,[42] D'Agostino et al.[43]

NGT and pre-T2DM health states

Three comorbidities are modelled in the NGT and pre-T2DM health states using equations developed from FHS data: MI,[42] CHF,[43] and stroke.[43] For each patient year, the transition equations for these three comorbidities are randomly ordered to allow events to occur in a random sequence throughout the year. The patient-specific probability of experiencing each of these comorbidities is then sequentially calculated by evaluating each of these equations using a patient's individual characteristics. Each probability is compared with a random number drawn from a uniform distribution to determine whether each comorbidity occurs for each patient.

The FHS equations for CHF and stroke use a Cox-proportional hazard model with a baseline survival function for 10-year survival. An exponential distribution for the baseline hazard function (constant over time) was assumed to calculate the baseline survival function for one-year survival. Only the first occurrence of MI and stroke are modelled in the NGT and pre-T2DM health states, as that is an underlying assumption in the FHS transition equations. If a patient starts the simulation with a history of MI or stroke, that type of event cannot occur again in the NGT or pre-T2DM health state. If the patient transitions to the T2DM health state, a second occurrence of that type of event can occur.

Amputation, renal failure and blindness are not modelled in the NGT or pre-T2DM health states, as no usable transition equations were found in a literature review. This is likely due to the strong association between these comorbidities and T2DM, where patients with T2DM have a 10-fold increased risk of lower extremity amputation[44] and a nine-fold increased risk of renal failure[45] compared to patients who do not have T2DM. As such, the model may slightly underestimate the overall occurrence rate of each of these comorbidities.

IHD is not modelled in the NGT or pre-T2DM health states due to discrepancies in the definition of IHD between the FHS and the UKPDS. Most notably, the FHS includes MI in the definition of IHD, whereas the UKPDS does not include MI. The FHS transition equation for IHD includes an independent variable for T2DM status; as such, it could be used to model the risk of IHD in both T2DM and non-T2DM health states, however given that UKPDS-OM2 transition equations are used for all other parameters in the T2DM health state, the preference is to use the UKPDS-OM2 equation for IHD in the T2DM health state and exclude IHD from the NGT and pre-T2DM health states as MI is already modelled separately.

T2DM health state

Eight comorbidities are modelled in the T2DM health state using the UKPDS-OM2 transition equations: CHF, IHD, blindness, renal failure, MI, stroke, amputation, and diabetic ulcer. For each patient year, the transition equations for these eight comorbidities are randomly ordered to allow events to occur in a random sequence throughout the year. The patient-specific probability of experiencing each of these comorbidities is then sequentially calculated by evaluating each of these equations using the patient's individual characteristics. Each probability is compared with a random number is drawn from a uniform distribution to determine whether each comorbidity occurs for each patient. If a comorbidity is predicted to occur, it will inform the remaining set of equations that are still to be estimated in the same year.[3]

CHF, IHD, blindness, and renal failure are considered chronic complications. Once a patient experiences one of these comorbidities, they continue to experience it for the remainder of the simulation or until they die. The transition equation for that specific comorbidity type is no longer evaluated in subsequent years. If a patient starts the simulation with one of these comorbidities, they also continue to experience it for the remainder of the simulation or until they die.

MI, stroke, and amputation are considered acute events, a patient can experience each of these events up to two times, however they cannot experience the same type of event twice in the same year. This is an underlying assumption in the UKPDS-OM2 equations. The first and second occurrences of MI, stroke, and amputation use different transition equations. Once a patient experiences their first occurrence of one of these types of events, the first occurrence transition equation for that type of event is no longer evaluated in subsequent years. In the following years, the second occurrence transition equation is evaluated until the event occurs, the simulation ends, or the individual dies.

The transition equation for diabetic ulcer is evaluated for each patient in each year to determine if the patient experiences an ulcer in the given year regardless of the patient's history of ulcer.

Mortality Transitions

The following section describes the mortality transition equations used in the second level of the model, which are conditioned on a patient's T2DM health states as determined in the top level of the model. The variables included in the mortality transition equations/parameters are displayed in Table 8.

TABLE 8: Variables in mortality transition equations

Variable	T2DM mortality				non-T2DM mortality			
	Eq. 1	Eq. 2	Eq. 3	Eq. 4	CHF	MI	Stroke	All-other-cause
**Age**		t		t		t	t	t
**Age at T2DM diagnosis**		t						
**Sex**	t					t	t	t
**Ethnicity**		t						
**Smoke**	t-1	t-1	t-1					
**BMI**			t-1					
**HDL**				t-1				
**HR**		t-1						
**WBC**			t-1	t-1				
**MMALB**			t-1					
**PVD**		t-1		t-1				
**AF**				t-1				
**CHF**			t-1		t			
**IHD**		t		t, t-1				
**Renal failure**		t	t-1	t-1				
**MI**		t		t, t-1		t		
**Stroke**		t	t-1	t			t	
**LEA**		t	t-1	t, t-1				

Notes: 't' indicates time-invariant (i.e., sex) or time logic (age)/current period, 't-1' indicates any prior period; AF: atrial fibrillation; BMI: body mass index; CHF: congestive heart failure; HDL: high-density lipoprotein; HR: heart rate; IHD: ischemic heart disease; LEA: lower extremity amputation; MI: myocardial infarction; MMALB: micro- or macro-albuminuria; PVD: peripheral vascular disease; T2DM: type 2 diabetes mellitus; WBC: white blood cell count.
Sources: Hayes et al.,[3] Vemmos et al.,[46] AMI Statistics,[47] McAlister et al.,[48] Statistics Canada (2022)[49]

NGT and pre-T2DM health states

The probability of mortality in the NGT and pre-T2DM health states depends on a patient experiencing any of the three possible comorbidities (MI, CHF, or stroke) in a given year. If, in any patient year, one or more of these comorbidities are experienced, the one-year age and sex-specific all-cause mortality probability for each comorbidity type are applied in the order in

which the comorbidities occur. The general all-other-causes mortality probability is applied if a patient does not experience any comorbidities in a given year. Details and sources for the morality equations are shown in Table 9, and the probability values can be found in Table 10. The overarching principle is that mortality of any cause is higher within the first year of experiencing an event. The UKPDS-OM2 mortality equations in the T2DM health state use a similar approach but combine all T2DM-related comorbidities (except blindness and ulcer) rather than having separate equations for each comorbidity type. No usable transition equations or probabilities were found in the literature review for one-year all-cause mortality by specific comorbidity type for non-T2DM individuals only. Therefore, the mortality probabilities given in Table 10 do not distinguish between individuals with and without T2DM.

The annual probability of death for all other causes is calculated from the annual mortality rate using the formula p=1-exp(-r). We exclude deaths resulting directly from MI, CHF, and stroke from the annual probability of death for all other causes. However, due to limitations in the data, we were unable to exclude all deaths within one year of these events. As the event-specific mortality probabilities for MI, CHF, and stroke include death from any cause within one year of the event occurring, there will be a small amount of overlap between the event-specific mortality probabilities and the all-other-causes mortality probability. Therefore, our model may overestimate mortality.

Table 9: NGT and pre-T2DM mortality equations

Comorbidity Type	Description	Population	Time frame and stratification	Source
Myocardial infarction	Proportion of deaths within one year of myocardial infection among all incident cases	Sweden, adults age 20 or older	2016-2020 (five-year average) by age and sex	AMI Statistics (2022)[47]
Congestive heart failure	Proportion of deaths within one year of hospital admission for congestive heart failure	Alberta, adults	2004-2008 (five-year average)	McAlister et al. (2013)[48]
Stroke	One year crude mortality (%) after first-ever stroke	Southern Greece, adults age 20 or older	Combined Nov. 1993 to Oct. 1995 by age and sex	Vemmos et al. (2000)[46]
All-other-causes	Annual probability of death from all other causes	Canada, adults age 20 or older	2016-2020 (five-year average) by age and sex	Statistics Canada (2022)[49]

TABLE 10: Mortality probabilities by comorbidity type for non-T2DM

Comorbidity and data source	Age group	Annual probability of mortality
**CHF**<br>Source: McAlister et al. (2013)[48]	All	0.34560
**MI**<br>Source: AMI Statistics (2022)[47]	**Male**	
	20-49	0.08600
	50-54	0.11300
	55-59	0.12800
	60-64	0.15700
	65-69	0.20500
	70-74	0.24700
	75-79	0.31600
	80-84	0.41100
	85+	0.58700
	**Female**	
	20-49	0.10700
	50-54	0.10400
	55-59	0.14700
	60-64	0.18800
	65-69	0.21000
	70-74	0.26100
	75-79	0.30400
	80-84	0.37800
	85+	0.56000
**Stroke**<br>Source: Vemmos et al. (2000)[46]	**Male**	
	18–54	0.16700
	55–64	0.19400
	65–74	0.24300
	75–84	0.37700
	85+	0.50700
	**Female**	
	18–54	0.22200
	55–64	0.24100
	65–74	0.32700
	75–84	0.40400
	85+	0.58500
**All-other-causes**	**Male**	
	20-24	0.00079

Source: Statistics Canada (2022)[49]	25-34	0.00010
	35-39	0.00126
	40-44	0.00157
	45-49	0.00219
	50-54	0.00331
	55-59	0.00516
	60-64	0.00803
	65-69	0.01221
	70-74	0.01877
	75-79	0.03032
	80-84	0.05151
	85-89	0.09013
	90+	0.16764
	**Female**	
	20-24	0.00035
	25-34	0.00042
	35-39	0.00052
	40-44	0.00067
	45-49	0.00137
	50-54	0.00219
	55-59	0.00343
	60-64	0.00524
	65-69	0.00797
	70-74	0.01272
	75-79	0.02089
	80-84	0.03631
	85-89	0.06510
	90+	0.14130

Sources: Vemmos et al.,[46] AMI Statistics,[47] McAlister et al.,[48] Statistics Canada (2022)[49]
Notes: CHF: congestive heart failure; MI: myocardial infarction

T2DM health state

Transition equations for mortality in the T2DM state are sourced from the UKPDS-OM2. The transition equations calculate the patient-specific probability of all-cause mortality each year, taking into account the patient's history of comorbidities and the onset of comorbidities the patient experiences in the current year (if any). A patient is considered to have a history of T2DM-related comorbidities if they have experienced any comorbidity at least once in prior

years. This includes chronic conditions that they may still be experiencing, such as CHF, as well as acute events, such as MI. All comorbidities except blindness and ulcer are associated with mortality in the year of onset, as per the UKPDS-OM2.[3] In addition, the mortality risk equations take into account various combinations of individual characteristics (age, sex, smoking status) and biomarkers (BMI, HDL, heart rate) as outlined in Table 8, above. In any patient-year, the patient-specific probability of mortality is calculated by evaluating one of four equations using a patient's individual characteristics. The four mutually-exclusive equations are defined for:

- Patients with no history of T2DM-related comorbidities and who have not experienced the onset of any T2DM-related comorbidities (except blindness or ulcer) in the current year.
- Patients with no history of T2DM-related comorbidities but experience the onset of one or more T2DM-related comorbidities (expect blindness or ulcer) in the current year.
- Patients with a history of T2DM-related comorbidities who have not experienced the onset of any T2DM-related comorbidities (except blindness or ulcer) in the current year.
- Patients with a history of T2DM-related comorbidities experience the onset of one or more T2DM-related comorbidities (expect blindness or ulcer) in the current year.

The calculated probability of mortality is then compared with a random number is drawn from a uniform distribution to determine if the patient dies.

Risk Factor Transitions

Individual characteristics, biomarkers, and the presence of certain clinical conditions (for example, atrial fibrillation) are risk factors in predicting T2DM, T2DM-related complications, and death. In theory, modelling changes in clinical risk factors over time using risk factor transition equations should improve the modelling of long-term health outcomes.[11] This model uses a patient's risk factors from the previous year to update their risk factors in the current year with risk factor transition equations. The risk factor transition equations consider the effect of aging (time), biomarker interactions, and the presence of disease (particularly T2DM) on the change in risk factors over time to simulate natural disease progression and aging in the population. The specific combinations of independent variables used in each risk factor transition equation can be seen in Table 11.

TABLE 11: Variables in risk factor transition equations

Variables	T2DM risk factor trajectories													non-T2DM risk factor trajectories						
	Smoker	BMI	eGFR	HAEM	HbA1c	HDL	LDL	HR	SBP	WBC	MMALB	PVD	AF	BMI	HbA1c	TCHOL	HDL	SBP	LVH	HYP-TRT
**Age**	t	t	t	t	t		t	t	t	t				t		t	t	t	t	t
**Age at T2DM diagnosis**	t	t	t	t	t		t	t	t	t	t	t	t							
**Sex**	t	t	t	t	t	t	t	t	t	t	t			t	t	t	t	t	t	t
**Ethnicity**		t	t	t	t	t	t		t	t					t			t	t	
**Smoke**	t-1										t-1	t-1			t-1			t-1		
**BMI**		t-1	t-1								t-1	t-1	t-1	t-1	t-1	t-1	t-1	t-1	t-1	
**eGFR**			t-1																	
**HAEM**				t-1																
**HbA1c**					t-1						t-1	t-1			t-1					
**TCHOL**																t-1				
**HDL**			t-1			t-1					t-1						t-1			
**LDL**			t-1				t-1					t-1								
**HR**								t-1												
**SBP**			t-1						t-1		t-1	t-1						t-1	t-1	t-1
**DBP**																			t-1	t-1
**WBC**										t-1										
**FT2DM**												t			t					
**FCVD**														t				t		

Sources: Leal et al.,[11] Breeze et al.[10] *Notes:* 't' indicates time-invariant (i.e., sex) or time logic (age)/current period, 't-1' indicates any prior period; BMI: body mass index; DBP: diastolic blood pressure; eGFR: estimated glomerular filtration rate; FCVD: family history of cardiovascular disease; FT2DM: family history of type 2 diabetes mellitus; HAEM: hemoglobin; HbA1c: glycate hemoglobin; HDL: high-density lipoprotein; HR: heart rate; LDL: low-density lipoprotein; SBP: systolic blood pressure; TCHOL: total cholesterol; T2DM: type 2 diabetes mellitus; WBC: white blood cell count

NGT and pre-T2DM health states

All risk factors that are used in the NGT and pre-T2DM comorbidity transition equations are updated annually using risk factor transition equations developed by Breeze et al. (2016)[10] except for the presence of left ventricle hypertrophy (LVH), the presence of anti-hypertensive treatment, diastolic blood pressure (DBP), and low-density lipoprotein (LDL). The risk factor transition equations developed by Breeze et al. (2016) use 16 years of longitudinal data from a cohort of civil servants in the United Kingdom (Whitehall cohort II) and apply latent growth curve modelling to estimate trajectories for multiple risk factors simultaneously.

The prediction equation for LVH comes from an analysis by de Simone et al. (1994), which developed logistic regression equations to estimate the probability of LVH for males and females separately, using SBP, DBP, age, BMI, and race as predictors.[23]

The probability of an individual receiving anti-hypertensive treatment is derived from the Canadian Health Measures Survey (combined 2012 to 2015). The survey results show the percent of hypertensive respondents (SBP greater than or equal to 140 or DBP greater than or equal to 90) receiving anti-hypertensive treatment by age group.[50] In our model, once patients receive anti-hypertensive treatment, they continue to receive it for the remainder of the simulation. We also assume that anti-hypertensive treatment is effectively able to manage hypertension, and therefore, SBP remains stable over time once a patient is treated.

We were unable to find usable equations in the literature for DBP and LDL. DBP is assumed to be constant and does not change over time. DBP is only an independent variable in the LVH equation in the NGT and pre-T2DM health states. It is not used in any equations in the T2DM health state. LDL is calculated based on the patient's total cholesterol, HDL, and triglycerides. Triglycerides are assumed to be constant over time, but total cholesterol and HDL are updated annually using risk factor transition equations developed by Breeze et al. (2016).

T2DM health state

Once a patient is in the T2DM health state, all risk factors used in the T2DM comorbidity and mortality transition equations are updated annually using the risk factor transition equations developed by UKPDS-OM2. These equations use 24 years of longitudinal data from the UKPDS clinical trial and subsequent follow-up. The UKPDS-OM2 risk factor transition equations are commonly used in other diabetes simulation models.[4, 11]

Intervention Effectiveness

The intervention effectiveness parameters in the model are from the intensive lifestyle intervention arm of the DPP using the outcomes published in the ten-year follow-up Diabetes Prevention Program Outcomes Study (DPPOS).[51] The intervention effect on weight change (converted to change in BMI) and HbA1c are reported each year following randomization into the treatment arm for ten years.[9] DPPOS also found that the incidence rate of T2DM in the intensive lifestyle intervention group was reduced by 34% compared to the placebo group over the ten-year period.[9] In our model, when the DPP impact on HbA1c and BMI were modelled alone, the expected reduction in T2DM incidence was not observed. The DPP diagnosed T2DM using FPG and OGTT instead of HbA1c, which is used in the model. Studies suggest HbA1c may not identify as many people as having T2DM compared to FPG or OGTT, which may explain the lower reduction in the incident rate of T2DM in our model.[52] To more closely reflect the T2DM incidence rate found in the DPP, the model was calibrated with an additional reduction in the probability of a sudden increase in HbA1c. Therefore, the mechanisms through which the intervention reduces the incidence or delays the progression of T2DM based on the DPPOS are through a reduction in the annual rate of change for (i) BMI and (ii) HbA1c and also (iii) the calibration factor on the probability of a sudden increase in HbA1c. As there is only evidence showing the effects of an intensive lifestyle intervention for the first ten years after randomization, following this period, no further intervention effects are assumed. Patients are assumed to continue on a trajectory consistent with natural disease progression in the absence of the intervention based on their health status at the end of the 10-year period following treatment. This is a conservative assumption based on the lack of evidence for longer-term effects.

In the model, the effects on BMI and HbA1c are modelled directly, while the effects on blood pressure, cholesterol, and other risk factors are modelled indirectly through the treatment impact on BMI. For each patient in the treatment arm of the model, BMI and HbA1c levels are updated annually using the results from DDP/DPPOS given in Table 12 as follows:

1. The model calculates what the patient’s HbA1c and BMI would be if they had not been enrolled in the intervention using the appropriate risk factor transition equations.
2. The model then applies the intervention effects from Table 12 to the patient’s HbA1c and BMI calculated in step 1 for each year in the 10-year period following treatment. This

occurs irrespective of the patient's T2DM health state. If an individual transitions to T2DM, the effects of the intervention on HbA1c and BMI continue to be applied.

3. For patients with an update HbA1c of 6.0 to 6.4, a 34% reduction is applied to their probability of experiencing a sudden increase in HbA1c and transitions to T2DM.
4. After the first ten years following treatment, no further intervention effects are assumed, and the patient's HbA1c and BMI continue on trajectories consistent with natural disease progression based on their HbA1c and BMI at the end of the 10-year period following treatment.

Steps one through four are visually displayed for HbA1c in Figure 2 and for BMI in Figure 3.

FIGURE 2: Intervention impacts on HbA1c

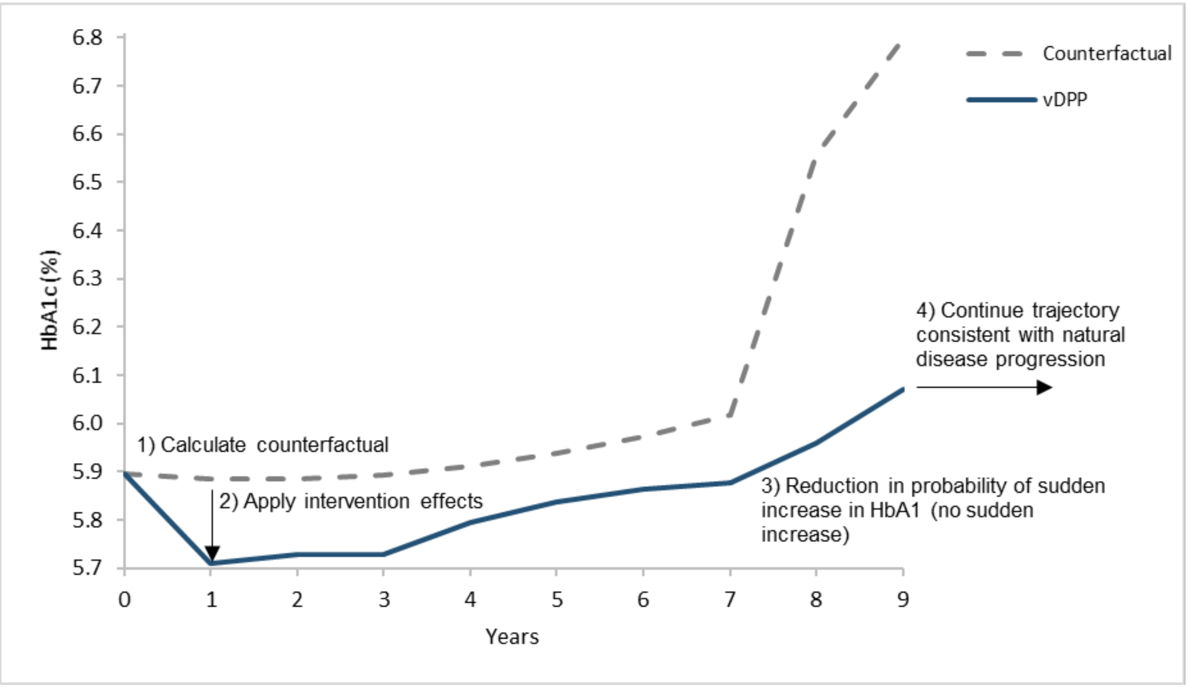

FIGURE 3: Intervention impacts on BMI

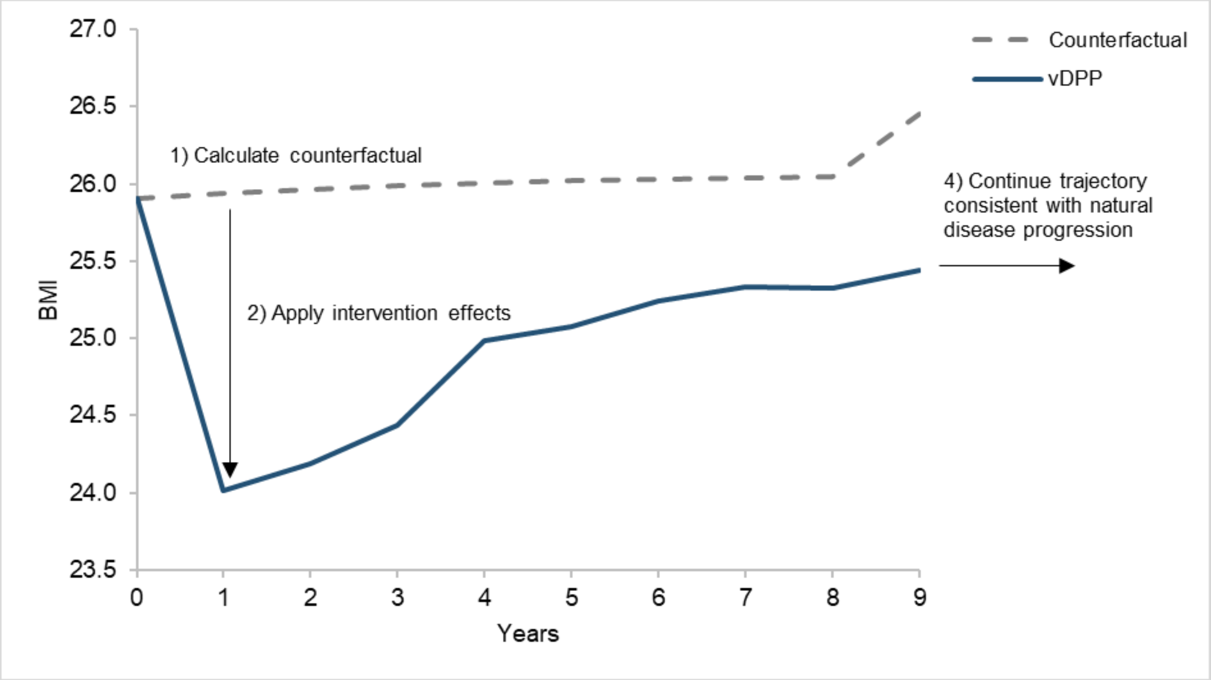

Note: Step 3) is not included on this graph as it does not apply to BMI. The modelled percent reduction in BMI is equivalent to the percent reduction in weight when assuming a patient's height is constant.

TABLE 12: DPP/DPPOS effectiveness for intensive lifestyle intervention arm compared to the placebo arm

Years since DPP randomization	Percent reduction in BMI			Percent reduction in HbA1c	Percent reduction in the probability of a sudden increase in HbA1c
	Age 25-44	Age 45-59	Age 60+	All age groups	All age groups
1	6.93	6.84	7.41	3.00	34
2	5.32	5.76	6.82	2.65	
3	4.69	4.42	5.92	2.81	
4	3.21	2.67	3.91	1.99	
5	1.61	1.48	3.61	1.67	
6	1.31	1.63	3.01	1.83	
7	1.68	1.92	2.72	2.33	
8	2.02	1.63	2.72	1.83	
9	1.31	1.19	1.99	1.00	
10	1.31	0.89	0.31	0.83	

Notes: The modelled percent reduction in BMI is equivalent to the percent reduction in weight when assuming a patient's height is constant. BMI: body mass index; HbA1c: glycated hemoglobin; T2DM: type 2 diabetes mellitus; Source*:* Diabetes Prevention Program Research Group et al. [9]

Intervention compliance

Intervention compliance is evaluated for each patient at the start of the simulation, and a patient is determined to be either compliant or not compliant. The CDC accreditation process defines compliance as a patient completing at least eight sessions in months one through six of the program and whose time from the first session to the last session attended is at least nine months (i.e., completers).[53] If a patient is determined to be not compliant, no intervention effects are assumed, and the patient follows the same trajectory of disease progression as in standard care.

The treatment compliance rate in the model is assumed to be 50%. The definitions used to measure compliance and/or retention vary between studies. In addition, retention can vary based on differing implementation strategies. We base our compliance rate on retention and participation in the CDC National DPP in the United States.[54-56]

1.4 Validation

Through multiple iterations of model development, pressure testing, and extreme value analysis, we verified that all model elements were implemented correctly and that the model generated logical results. We conducted a series of validation checks to ensure the UKPDS-OM equations used in the model equations were implemented correctly. They are as follows:

- Each comorbidity type in the model is only allowed to occur once. If a patient starts the simulation with a history of a comorbidity, that type of event cannot occur again. We ran a simulation where all patients began the simulation with a history of all comorbidities and verified that no comorbidities occurred during the simulation (i.e., the model was not allowing comorbidities to occur more than once).
- We also ran the simulation with the simulation population outlined in Supplemental Material Section 2. We confirmed that the sum of each type of comorbidity for each patient did not exceed one to ensure the model was not double-counting events.
- We confirm that all probabilities calculated within the model were only sampled once per patient per cycle.
- We confirmed that correct equations were being evaluated for the patient's health state (i.e., T2DM, pre-T2DM, NGT).
- We confirmed that once a patient dies, the model does not allow them to experience additional comorbidities.
- We conducted a comparison to ensure the alignment between the average probability of each comorbidity occurring in each cycle, as calculated by the risk equations in the model, and the total number of events observed in each cycle.

In addition to the validation described above, we also conducted a series of validation checks to ensure that all model elements were implemented correctly and that the model generated logical results. Some examples include:

- We compared the simulated annual event rate for MI, CHF, stroke, and death for patients with and without T2DM to ensure the event rates were lower in the latter group.

- We ensured our simulation did not generate negative biomarker values (ex., negative BMI).
- We set the intervention effectiveness parameters to zero and ensure the results are the same as the results for standard care.
- We ensured the simulated QALYs never exceeded one for any patient in any time cycle.
- We conducted a series of checks, setting specific costs in the model to extreme values (e.g., zero or 1,000,000) to ensure the model generates results that align with these extremes.

We performed an internal validation of the T2DM health state to examine how well the model predicts the outcomes in the data set used to derive the UKPDS-OM2 equations. We conducted an external validation to determine how well the model predicts T2DM occurrence in a data set that was not used to derive the equations or parameters in the model. The methods for both validation exercises are described in the main text.

1.5 Case study analysis: Cost-effectiveness of a hypothetical diabetes prevention program

This economic analysis evaluates the cost-effectiveness of a hypothetical intensive lifestyle intervention based on the DPP compared to Alberta's current standard of care. The current standard of care varies; however, it typically consists of primary care providers recommending healthy behaviour changes and offering standard lifestyle recommendations during regularly scheduled appointments.[38, 57]

The model uses a one-year cycle length, a 15-year time horizon, and a discount rate of 1.5% for both costs and QALYs as per CDA guidelines.[29] The analysis follows the perspective of the healthcare system, where only costs incurred by the healthcare payer are included. The primary outcome of this cost-effectiveness analysis is the QALY, a composite measure of length and quality of life. The QALY is particularly valuable for economic evaluations as it has a threshold from which it can be judged whether an intervention is cost-effective compared to a comparator. Additional outcomes in this analysis include the event rate of incident T2DM and the event rate of microvascular, neuropathic, and cardiovascular complications and deaths.

The case study results are summary findings of a probabilistic sensitivity analysis (PSA) simulating 5,100 patients in each arm (standard care and intervention). A PSA was performed to

reflect the uncertainty in the model input parameters on the outcomes. The model was run for 1,000 simulations (over all patients in each arm), sampling from the underlying probability distributions for the input parameters, as indicated in the following PSA subsection. By repeating the process of drawing from the parameter probability distributions and capturing the model outcomes, the range of values parameters are likely to take was represented in the range of model outputs. The average per-patient costs and QALYs are reported, along with 95% Uncertainty Intervals [UI]. In addition, the expected net monetary benefit (NMB) at a WTP threshold of $30,000 is estimated for each strategy, reflecting health system opportunity costs estimated for Canada.[58] For the intervention, the incremental net monetary benefit (INMB) is estimated relative to standard care for each year over the 15-year model time horizon to determine the time point at which the intervention becomes cost-effective. In addition, the cumulative T2DM event rate and the event rate for each comorbidity type over the 15-year model time horizon are presented for both standard care and the intervention. The results of the PSA are reported in the form of a cost-effectiveness plane and a density plot of the NMB for each strategy.

Probabilistic sensitivity analysis

Table 13 below displays the mean, standard error, and underlying distribution for the model parameters included in the PSA. No variance measures were available in the source literature for the percent reduction in BMI with the intervention or for the costs. We assumed the standard error to be 10% of the mean value for these parameters. In addition, Leal et al. (2021)[11] provided 4,000 sets of fully correlated regression coefficients for all UKPDS risk factor transition equations in their supplementary material. For each PSA iteration, we obtain parameter values for the UKPDS risk factor transition equation coefficients by randomly sampling with replacement from these sets of correlated coefficients. We excluded the coefficients for the comorbidity transition equations from the PSA. We propagated uncertainty in the probability of comorbidities occurring by considering the uncertainty in each individual risk factor, which serves as independent variables in these equations.

TABLE 13: Variables included in the probabilistic sensitivity analysis

Parameter description	Mean value	SE value	Distribution	Source
**Probabilities**				
Probability of death from CHF non-T2DM	0.345600	0.00365	beta	McAlister et al. (2013)[48]
Probability of death from stroke non-T2DM for males aged 20-54	0.167000	0.08550	beta	Vemmos et al. (2000)[46]
Probability of death from stroke for non-T2DM males aged 55-64	0.194000	0.06506	beta	
Probability of death from stroke for non-T2DM males aged 65-74	0.243000	0.04954	beta	
Probability of death from stroke for non-T2DM males aged 75-84	0.377000	0.04686	beta	
Probability of death from stroke for non-T2DM male age 85-114	0.507000	0.05735	beta	
Probability of death from stroke for non-T2DM female age 20-54	0.222000	0.13147	beta	
Probability of death from stroke for non-T2DM females aged 55-64	0.241000	0.07813	beta	
Probability of death from stroke for non-T2DM females aged 65-74	0.327000	0.06270	beta	
Probability of death from stroke for non-T2DM females aged 75-84	0.404000	0.04907	beta	
Probability of death from stroke for non-T2DM female age 85-114	0.584000	0.06705	beta	
Probability of death from MI for male non-T2DM age 20-49	0.086000	0.01387	beta	AMI Statistics (2022)[47]
Probability of death from MI for male non-T2DM age 50-54	0.113000	0.01226	beta	
Probability of death from MI for male non-T2DM age 55-59	0.128000	0.01268	beta	
Probability of death from MI for male non-T2DM age 60-64	0.157000	0.02017	beta	
Probability of death from MI for male non-T2DM age 65-69	0.205000	0.01218	beta	
Probability of death from MI for male non-T2DM age 70-74	0.247000	0.01135	beta	
Probability of death from MI for male non-T2DM age 75-79	0.316000	0.01387	beta	
Probability of death from MI for male non-T2DM age 80-84	0.411000	0.01481	beta	

Probability of death from MI for male non-T2DM age 85-114	0.587000	0.01024	beta	
Probability of death from MI for female non-T2DM age 20-49	0.107000	0.02317	beta	
Probability of death from MI for female non-T2DM age 50-54	0.104000	0.00846	beta	
Probability of death from MI for female non-T2DM age 55-59	0.147000	0.02526	beta	
Probability of death from MI for female non-T2DM age 60-64	0.188000	0.02764	beta	
Probability of death from MI for female non-T2DM age 65-69	0.210000	0.01909	beta	
Probability of death from MI for female non-T2DM age 70-74	0.261000	0.01778	beta	
Probability of death from MI for female non-T2DM age 75-79	0.304000	0.01405	beta	
Probability of death from MI for female non-T2DM age 80-84	0.378000	0.02003	beta	
Probability of death from MI for female non-T2DM age 85-114	0.560000	0.01696	beta	
Probability of death from all other causes for male non-T2DM age 20-24	0.000787	0.00004	beta	Statistics Canada (2022)[49]
Probability of death from all other causes for male non-T2DM age 25-29	0.000999	0.00011	beta	
Probability of death from all other causes for male non-T2DM age 30-34	0.001120	0.00012	beta	
Probability of death from all other causes for male non-T2DM age 35-39	0.001260	0.00015	beta	
Probability of death from all other causes for male non-T2DM age 40-44	0.001570	0.00018	beta	
Probability of death from all other causes for male non-T2DM age 45-49	0.002190	0.00015	beta	
Probability of death from all other causes for male non-T2DM age 50-54	0.003310	0.00014	beta	
Probability of death from all other causes for male non-T2DM age 55-59	0.005160	0.00017	beta	
Probability of death from all other causes for male non-T2DM age 60-64	0.008030	0.00017	beta	
Probability of death from all other causes for male non-T2DM age 65-69	0.012210	0.00023	beta	
Probability of death from all other causes for male non-T2DM age 70-74	0.018770	0.00027	beta	
Probability of death from all other causes for male non-T2DM age 75-79	0.030320	0.00062	beta	
Probability of death from all other causes for male non-T2DM age 80-84	0.051510	0.00095	beta	

Probability of death from all other causes for male non-T2DM age 85-89	0.090130	0.00227	beta	
Probability of death from all other causes for male non-T2DM age 90-114	0.167640	0.00433	beta	
Probability of death from all other causes for female non-T2DM age 20-24	0.000348	0.00004	beta	
Probability of death from all other causes for female non-T2DM age 25-29	0.000421	0.00005	beta	
Probability of death from all other causes for female non-T2DM age 30-34	0.000522	0.00005	beta	
Probability of death from all other causes for female non-T2DM age 35-39	0.000662	0.00005	beta	
Probability of death from all other causes for female non-T2DM age 40-44	0.000898	0.00004	beta	
Probability of death from all other causes for female non-T2DM age 45-49	0.001372	0.00005	beta	
Probability of death from all other causes for female non-T2DM age 50-54	0.002190	0.00008	beta	
Probability of death from all other causes for female non-T2DM age 55-59	0.003430	0.00009	beta	
Probability of death from all other causes for female non-T2DM age 60-64	0.005239	0.00015	beta	
Probability of death from all other causes for female non-T2DM age 65-69	0.007970	0.00014	beta	
Probability of death from all other causes for female non-T2DM age 70-74	0.012720	0.00028	beta	
Probability of death from all other causes for female non-T2DM age 75-79	0.020890	0.00034	beta	
Probability of death from all other causes for female non-T2DM age 80-84	0.036310	0.00065	beta	
Probability of death from all other causes for female non-T2DM age 85-89	0.065100	0.00227	beta	
Probability of death from all other causes for female non-T2DM age 90-114	0.141290	0.00571	beta	
Probability of being on hypertensive treatment for males aged 20-39	0.475000	0.09790	beta	DeGuire et al. (2019) [50]
Probability of being on hypertensive treatment for males aged 40-59	0.705000	0.03660	beta	
Probability of being on hypertensive treatment for males aged 60-69	0.862000	0.01970	beta	
Probability of being on hypertensive treatment for males age 70+	0.911000	0.01970	beta	
Probability of being on hypertensive treatment for females age 20-39	0.652000	0.09720	beta	
Probability of being on hypertensive treatment for females aged 40-59	0.748000	0.03610	beta	

Probability of being on hypertensive treatment for females aged 60-69	0.838000	0.02330	beta	
Probability of being on hypertensive treatment for females age 70+	0.864000	0.02230	beta	
**UKPDS risk factor transition equations**				
Sampled from sets of fully correlated coefficient values	-	-	-	Leal et al. (2021)[11]
**Breeze risk factor transition equations**				
**BMI equation**				Breeze et al. (2016)[10]
Linear slope constant term	0.6409	0.0420	normal	
Linear slope - Beta coefficient - age	-0.0084	0.0010	normal	
Linear slope - Beta coefficient - sex	-0.0285	0.0110	normal	
Linear slope - Beta coefficient - family history of cardiovascular disease	-0.0155	0.0000	normal	
Quadratic slope constant term	-0.2007	0.0230	normal	
Quadratic slope - Beta coefficient - age	0.0026	0.0009	normal	
Quadratic slope - Beta coefficient - sex	0.0089	0.0060	normal	
Quadratic slope - Beta coefficient - family history of cardiovascular disease	0.0104	0.0060	normal	
**Glucose equation**				
Linear slope constant term	-0.4255	0.071	normal	
Linear slope - Beta coefficient - sex	0.1486	0.045	normal	
Linear slope - Beta coefficient - ethnicity	-0.0218	0.081	normal	
Linear slope - Beta coefficient - family history of T2DM	-0.0512	0.054	normal	
Linear slope - Beta coefficient - smoker	0.1796	0.066	normal	
Linear slope - Beta coefficient - association with BMI intercept	0.0820	0.024	normal	
Linear slope - Beta coefficient - association with BMI linear slope	0.1984	0.073	normal	
Linear slope constant term	0.1094	0.025	normal	

Linear slope - Beta coefficient - sex	-0.0855	0.027	normal	
Linear slope - Beta coefficient - ethnicity	0.0899	0.049	normal	
Linear slope - Beta coefficient - family history of T2DM	0.0633	0.033	normal	
Linear slope - Beta coefficient - smoker	-0.0390	0.040	normal	
**HbA1c equation**				
Constant term	4.4769	0.073	normal	
Beta coefficient - glucose	0.5074	0.016	normal	
Beta coefficient - age	0.0101	0.001	normal	
Beta coefficient - sex	-0.0457	0.001	normal	
Beta coefficient - ethnicity	0.1854	0.030	normal	
Beta coefficient - family history of T2DM	0.0563	0.020	normal	
**Total cholesterol equation**				
Linear slope constant term	2.1216	0.128	normal	
Linear slope - Beta coefficient - age	-0.0316	0.002	normal	
Linear slope - Beta coefficient - sex	-0.2677	0.026	normal	
Linear slope - Beta coefficient - association with BMI intercept	-0.4808	0.035	normal	
Linear slope - Beta coefficient - association with BMI linear slope	0.9802	0.108	normal	
**HDL equation**				
Linear slope constant term	0.1241	0.034	normal	
Linear slope - Beta coefficient - age	0.0020	0.001	normal	
Linear slope - Beta coefficient - sex	0.0041	0.007	normal	
Linear slope - Beta coefficient - association with BMI intercept	-0.0400	0.100	normal	
**SBP equation**				

Linear slope constant term	-0.0277	0.0210	normal	
Linear slope - Beta coefficient - age	0.0024	0.0009	normal	
Linear slope - Beta coefficient - sex	-0.0004	0.0040	normal	
Linear slope - Beta coefficient - smoker	0.0205	0.0050	normal	
Linear slope - Beta coefficient - ethnicity	0.0224	0.0070	normal	
Quadratic slope - Beta coefficient - family history of cardiovascular disease	-0.0013	0.0040	normal	
Linear slope - Beta coefficient - association with BMI intercept	-0.0396	0.0060	normal	
Linear slope - Beta coefficient - association with BMI linear slope	0.2325	0.0190	normal	
**Other risk factor transition equations**				
**Left ventricular hypertrophy equation for males**				
Beta coefficient - SBP	0.02	0.0084	normal	de Simone et al. (1994) [23]
Beta coefficient - DBP	0.03	0.0118	normal	
Beta coefficient - age	0.03	0.0124	normal	
Beta coefficient - BMI	0.18	0.0403	normal	
**Left ventricular hypertrophy equation for females**				
Beta coefficient - SBP	0.03	0.01012	normal	
Beta coefficient - age	0.04	0.02198	normal	
Beta coefficient - BMI	0.18	0.04070	normal	
Beta coefficient - ethnicity	-1.12	0.38930	normal	
**T2DM transition parameters**				
Value of sudden increase in HbA1 for those with pre-T2DM	0.5400	0.060	normal	Heianza et al. (2012a) [39]
Probability of experiencing a sudden increase in HbA1c for those with pre-T2DM	0.1292	0.012	beta	Heianza et al. (2012b) [40]

Intervention parameters				
Percent reduction in HbA1c in cycle 1	0.0300	0.00001	beta	Diabetes Prevention Program Research Group et al. [9]
Percent reduction in HbA1c in cycle 2	0.0265	0.00001	beta	
Percent reduction in HbA1c in cycle 3	0.0281	0.00001	beta	
Percent reduction in HbA1c in cycle 4	0.0199	0.00002	beta	
Percent reduction in HbA1c in cycle 5	0.0167	0.00002	beta	
Percent reduction in HbA1c in cycle 6	0.0183	0.00002	beta	
Percent reduction in HbA1c in cycle 7	0.0233	0.00005	beta	
Percent reduction in HbA1c in cycle 8	0.0183	0.00005	beta	
Percent reduction in HbA1c in cycle 9	0.0100	0.00005	beta	
Percent reduction in HbA1c in cycle 10	0.0083	0.00009	beta	
Percent reduction in BMI for age 25-44 in cycle 1	0.0693	0.0069*	beta	
Percent reduction in BMI for age 25-44 in cycle 2	0.0532	0.0053*	beta	
Percent reduction in BMI for age 25-44 in cycle 3	0.0469	0.0047*	beta	
Percent reduction in BMI for age 25-44 in cycle 4	0.0321	0.0032*	beta	
Percent reduction in BMI for age 25-44 in cycle 5	0.0161	0.0016*	beta	
Percent reduction in BMI for age 25-44 in cycle 6	0.0131	0.0013*	beta	
Percent reduction in BMI for age 25-44 in cycle 7	0.0168	0.0017*	beta	
Percent reduction in BMI for age 25-44 in cycle 8	0.0202	0.0020*	beta	
Percent reduction in BMI for age 25-44 in cycle 9	0.0131	0.0013*	beta	
Percent reduction in BMI for age 25-44 in cycle 10	0.0131	0.0013*	beta	
Percent reduction in BMI for age 45-59 in cycle 1	0.0684	0.0068*	beta	
Percent reduction in BMI for age 45-59 in cycle 2	0.0576	0.0058*	beta	

Percent reduction in BMI for age 45-59 in cycle 3	0.0442	0.0044*	beta	
Percent reduction in BMI for age 45-59 in cycle 4	0.0267	0.0027*	beta	
Percent reduction in BMI for age 45-59 in cycle 5	0.0148	0.0015*	beta	
Percent reduction in BMI for age 45-59 in cycle 6	0.0163	0.0016*	beta	
Percent reduction in BMI for age 45-59 in cycle 7	0.0182	0.0018*	beta	
Percent reduction in BMI for age 45-59 in cycle 8	0.0163	0.0016*	beta	
Percent reduction in BMI for age 45-59 in cycle 9	0.0119	0.0012*	beta	
Percent reduction in BMI for age 45-59 in cycle 10	0.0089	0.0009*	beta	
Percent reduction in BMI for age 60+ in cycle 1	0.0741	0.0074*	beta	
Percent reduction in BMI for age 60+ in cycle 2	0.0682	0.0068*	beta	
Percent reduction in BMI for age 60+ in cycle 3	0.0595	0.0060*	beta	
Percent reduction in BMI for age 60+ in cycle 4	0.0391	0.0039*	beta	
Percent reduction in BMI for age 60+ in cycle 5	0.0361	0.0036*	beta	
Percent reduction in BMI for age 60+ in cycle 6	0.0301	0.0030*	beta	
Percent reduction in BMI for age 60+ in cycle 7	0.0272	0.0027*	beta	
Percent reduction in BMI for age 60+ in cycle 8	0.0272	0.0027*	beta	
Percent reduction in BMI for age 60+ in cycle 9	0.0119	0.0012*	beta	
Percent reduction in BMI for age 60+ in cycle 10	0.0031	0.0003*	beta	
Percent reduction in the probability of experiencing a sudden increase in HbA1c for those with pre-T2DM	0.3400	0.0460	beta	
**Utilities**				
Baseline utility value for males aged 20-24	0.8900	0.0030	beta	Alberta PROMS and EQ-5D Research Support Unit[34]
Baseline utility value for males aged 25-44	0.8800	0.0000	beta	
Baseline utility value for males age 45-64	0.8300	0.0030	beta	

Baseline utility value for males age 65-74	0.8300	0.0030	beta	
Baseline utility value for males aged 75-114	0.8100	0.0030	beta	
Baseline utility value for females age 20-24	0.8700	0.0030	beta	
Baseline utility value for females age 25-44	0.8700	0.0000	beta	
Baseline utility value for females age 45-64	0.8200	0.0030	beta	
Baseline utility value for females age 65-74	0.8200	0.0050	beta	
Baseline utility value for females aged 75-114	0.7900	0.0030	beta	
Utility decrement associated with T2DM	0.0350	0.0004	gamma	Sullivan et al. (2005) [35]
Utility decrement for MI in non-T2DM states	0.0345	0.0010	gamma	Sullivan et al. (2005)[35]
Utility decrement for CHF in non-T2DM states	0.0546	0.0010	gamma	
Utility decrement for stroke in non-T2DM states	0.0483	0.0009	gamma	
Utility decrement for first year post-MI in T2DM state	0.0650	0.0300	gamma	Alva et al. (2014)[36]
Utility decrement for CHF in T2DM state	0.1010	0.0320	gamma	
Utility decrement for IHD in T2DM state	0.0280	0.0220	gamma	
Utility decrement for stroke in T2DM state	0.1650	0.0350	gamma	
Utility decrement for amputation in T2DM state	0.1720	0.0450	gamma	
Utility decrement for renal failure in T2DM state	0.1018	0.0472	gamma	O'Reilly et al. (2011)[37]
Utility decrement for ulcer in T2DM state	0.0317	0.0010	gamma	Sullivan et al. (2005)[35]
**Costs**				
Incremental cost of T2DM with no complications vs non-T2DM	$1,459	$146*	gamma	O'Reilly et al. (2006)[30]<br>Rosella et al. (2016)[33]

Cost of IHD for patients with T2DM in 1st year	$6,805	$681*	gamma	O'Reilly et al. (2006)[30]
Cost of IHD for patients with T2DM in subsequent years	$3,929	$393*	gamma	
Cost of MI for patients with T2DM in 1st year fatal	$11,402	$1,140*	gamma	
Cost of MI for patients with T2DM in 1st year non-fatal	$21,741	$2,174*	gamma	
Cost of MI for patients with T2DM in subsequent years	$3,400	$340*	gamma	
Cost of CHF for patients with T2DM in 1st year	$19,889	$1,989*	gamma	
Cost of CHF for patients with T2DM in subsequent years	$5,576	$558*	gamma	
Cost of stroke for patients with T2DM in 1st year fatal	$10,729	$1,073*	gamma	
Cost of stroke for patients with T2DM in 1st year non-fatal	$29,614	$2,961*	gamma	
Cost of stroke for patients with T2DM in subsequent years	$4,109	$411*	gamma	
Cost of amputation for patients with T2DM in 1st year	$45,938	$4,594*	gamma	
Cost of amputation for patients with T2DM in subsequent years	$6,292	$629*	gamma	
Cost of blindness for patients with T2DM in 1st year	$3,638	$364*	gamma	
Cost of blindness for patients with T2DM in subsequent years	$2,592	$259*	gamma	
Cost of renal failure for patients with T2DM in 1st year	$29,475	$2,948*	gamma	
Cost of renal failure for patients with T2DM in subsequent years	$13,378	$1,338*	gamma	
Cost of episodic diabetic foot ulcer for patients with T2DM	$3,143	$314*	gamma	O'Brien et al. (2003)[31]
Cost of MI for patients without T2DM in 1st year fatal	$11,179	$1,118*	gamma	O'Reilly et al. (2006)[30] Choi et al. (2021)[32]
Cost of MI for patients without T2DM in 1st year non-fatal	$21,315	$2,132*	gamma	
Cost of MI for patients without T2DM in subsequent years	$3,333	$333*	gamma	
Cost of CHF for patients without T2DM in 1st year	$19,499	$1,950*	gamma	
Cost of CHF for patients without T2DM in subsequent years	$5,467	$547*	gamma	
Cost of stroke for patients without T2DM in 1st year fatal	$9,016	$902*	gamma	

Cost of stroke for patients without T2DM in 1st year non-fatal	$24,885	$2,489*	gamma	
Cost of stroke for patients without T2DM in subsequent years	$3,453	$345*	gamma	

Notes: * indicates that no measure of variance was available in the source study. The standard error was assumed to be 10% of the mean value.

Value of information

A value of information (VoI) analysis has been conducted using the PSA results to provide an estimation of value, in terms of cost and health outcomes, of collecting more data/information on key model parameters. Typically, this is most useful where the output of an economic evaluation is uncertain, often due to underlying parameters. New information reducing uncertainty in the underlying parameters will increase the chance of the correct decision being made, where a correct decision is adopting a cost-effective intervention or not adopting interventions that are not cost-effective. The 'value' of this information is a function of how likely it is to enable a decision to be made or changed.

We have presented the expected value of perfect information (EVPI). EVPI is the price a decision-maker would be willing to pay to have perfect information, such that all decision uncertainty is removed. Although it is not possible to remove all decision uncertainty in the real world, attaching a value to the perfect information provides a reference point (upper bound) for thinking about the value of additional information. Some parameters will be more uncertain than others.

2. Supplemental Results

2.1 Validation results

Internal validation

Table 14 shows the simulated and observed KM cumulative failure probability for each diabetes-related complication and all-cause mortality at years five, 10, 15, and 20, along with the absolute difference. Foot ulcer is only included for years five and 10 as the UKPDS study does not provide KM cumulative failure probabilities beyond 10 years.

TABLE 14: Predicted versus observed Kaplan–Meier cumulative failure probability

Category	Outcome	Year	Kaplan–Meier cumulative failure probability		
			Observed	Simulated	Absolute difference
Macrovascular	**CHF**	5	0.010	0.010	0.000
	**CHF**	10	0.027	0.027	0.000
	**CHF**	15	0.054	0.048	-0.006
	**CHF**	20	0.083	0.076	-0.007
	**IHD**	5	0.034	0.030	-0.004
	**IHD**	10	0.071	0.076	0.005
	**IHD**	15	0.121	0.126	0.005
	**IHD**	20	0.189	0.178	-0.011
	**Stroke**	5	0.014	0.012	-0.002
	**Stroke**	10	0.042	0.036	-0.006
	**Stroke**	15	0.076	0.066	-0.010
	**Stroke**	20	0.123	0.102	-0.021
	**MI**	5	0.041	0.053	0.012
	**MI**	10	0.106	0.118	0.012
	**MI**	15	0.162	0.200	0.038
	**MI**	20	0.223	0.280	0.057
Microvascular	**Blindness**	5	0.012	0.015	0.003
	**Blindness**	10	0.025	0.033	0.008
	**Blindness**	15	0.046	0.050	0.004
	**Blindness**	20	0.067	0.074	0.007
	**Amputation**	5	0.004	0.002	-0.002
	**Amputation**	10	0.011	0.008	-0.003
	**Amputation**	15	0.024	0.020	-0.004
	**Amputation**	20	0.046	0.040	-0.006
	**Renal failure**	5	0.003	0.002	-0.001
	**Renal failure**	10	0.007	0.008	0.001
	**Renal failure**	15	0.019	0.014	-0.005
	**Renal failure**	20	0.026	0.032	0.006
	**Foot ulcer**	5	0.011	0.002	-0.009
	**Foot ulcer**	10	0.018	0.008	-0.010
Mortality	**Death**	5	0.043	0.045	0.002
	**Death**	10	0.123	0.133	0.010
	**Death**	15	0.252	0.255	0.003
	**Death**	20	0.432	0.406	-0.026

Notes: Simulated mean Kaplan–Meier cumulative failure probability of each endpoint is calculated from 1,000 PSA iterations of 5,100 patients. CHF: congestive heart failure; IHD: ischemic heart disease; MI: myocardial infarction.

The mean absolute difference between predicted and observed values was less than one percent across all comorbidities and mortality. The model predicted microvascular comorbidities slightly better than macrovascular, with mean absolute differences of 0.1% and 0.4%, respectively. Overall, our model showed strong predictive performance for T2DM-related comorbidities and mortality within the UKPDS cohort.

Figure 4 shows the annual mean of the simulated average risk factor progression for each risk factor with the average observed risk factor progression and 95% confidence interval from the UKPDS over a 20-year follow-up period.

FIGURE 4: Simulated and observed average risk factor progression

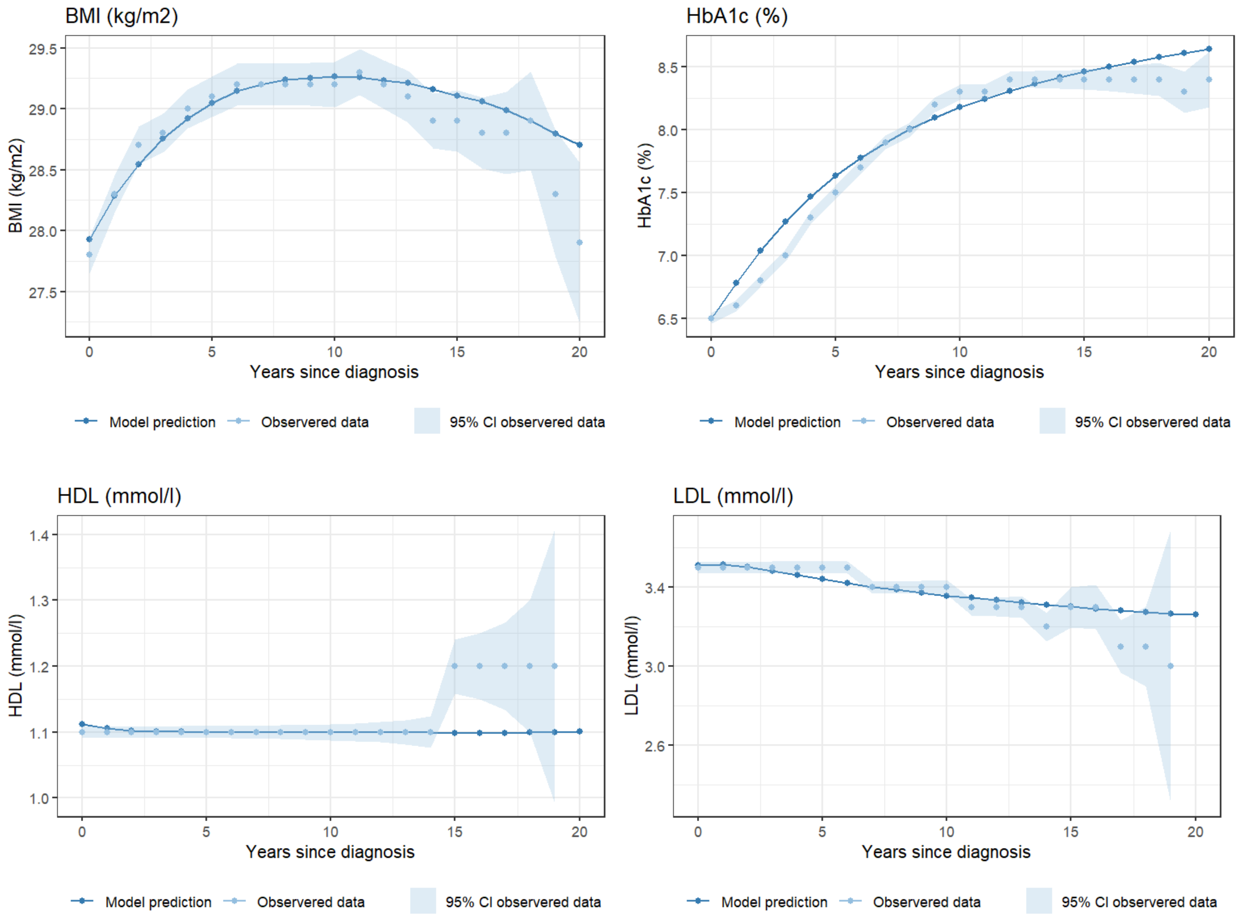

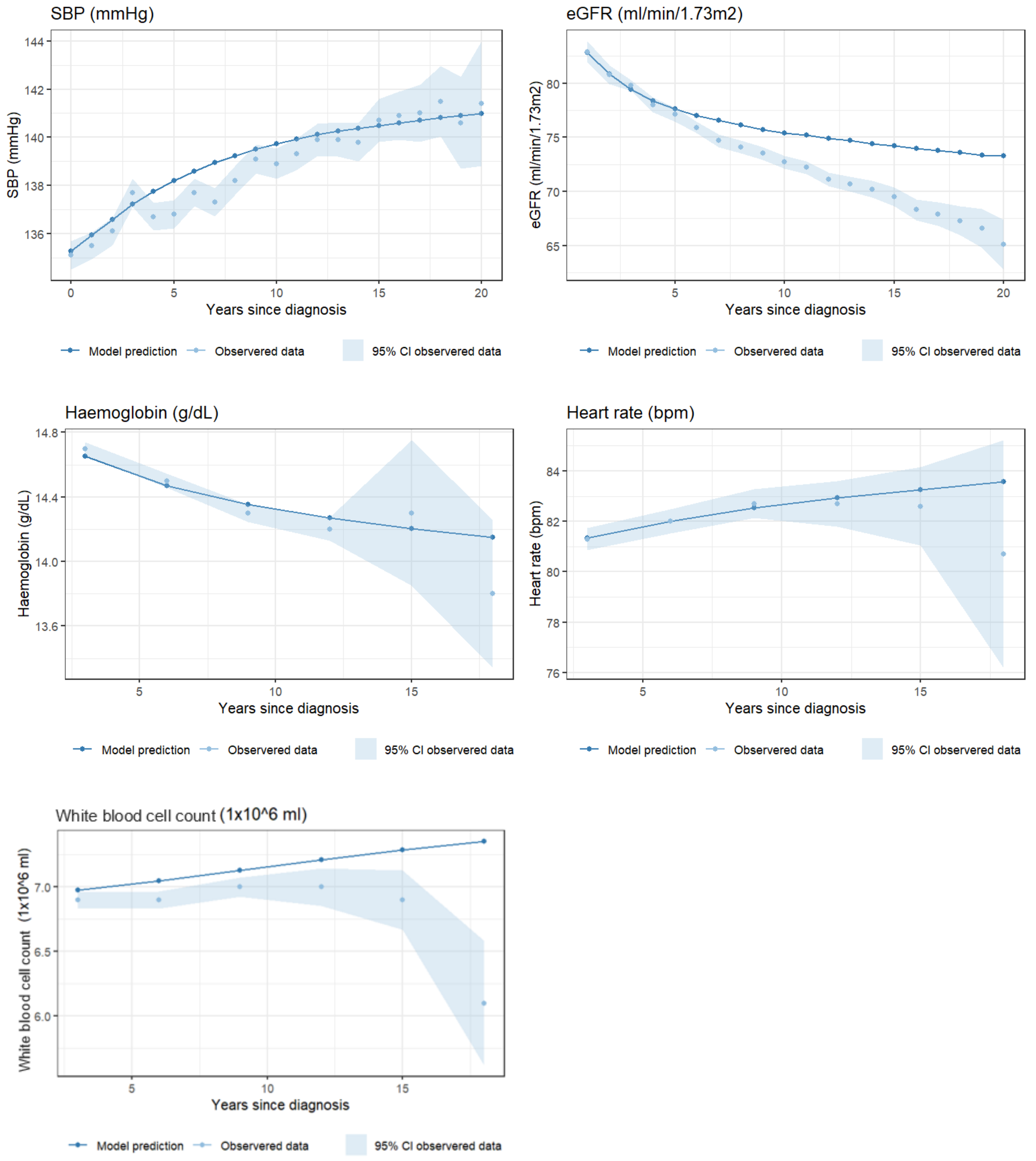

Notes: BMI: body mass index; CI: confidence interval; DBP: diastolic blood pressure; eGFR: estimated glomerular filtration rate; HbA1c: glycate hemoglobin; HDL: high-density lipoprotein; LDL: low-density lipoprotein; SBP: systolic blood pressure.

Overall, our model predicted risk factors within the UKPDS cohort reasonably well. Notably, our model predicted higher eGFR and white blood cell count than observed in the UKPDS. Ideally, when internally validating a model, we would use patient-specific data from the source study, or

if such data is unavailable, we would use data reflecting the distribution of patient characteristics and other variables and the correlation between these variables at the patient level. In this case, we did not have either patient-specific data or data on the correlation of initial patient characteristics. Therefore, we could only validate results using aggregate average values and non-correlated initial patient characteristics. This likely contributes to the discrepancy between the simulated and observed risk factor progression.

External validation

Table 15 shows the mean simulated and observed KM cumulative incidence of T2DM since randomization into the DPP annually for 15 years for the intensive lifestyle intervention arm and placebo arm, along with the simulated to observed ratio.

TABLE 15: Predicted versus observed Kaplan–Meier cumulative incidence of T2DM

Year	Intensive lifestyle intervention			Placebo		
	Observed (%)	Simulated (%)	Simulated/ Observed	Observed (%)	Simulated (%)	Simulate/ Observed
1	3.61	4.26	1.18	12.51	6.07	0.48
2	7.62	7.27	0.95	22.31	11.26	0.5
3	13.03	10.46	0.8	29.49	16.55	0.56
4	17.97	13.48	0.75	33.8	21.58	0.64
5	23.28	17.26	0.74	37.26	26.2	0.7
6	28.7	21.21	0.74	40.1	30.88	0.77
7	31.97	24.74	0.77	43.43	35.8	0.82
8	34.99	29.95	0.86	46.52	40.88	0.88
9	37.48	36.47	0.97	49.75	46.06	0.93
10	41.19	42.87	1.04	52.37	51.85	0.99
11	43.74	49.42	1.13	54.19	57.67	1.06
12	47.61	56.26	1.18	56.46	63.53	1.13
13	50.13	63.16	1.26	58.76	69.53	1.18
14	52.53	69.62	1.33	59.89	75.14	1.25
15	54.39	75.27	1.38	61.59	79.9	1.3

Simulated to observed ratios close to one suggest that, on average, the model produces estimates close to the observed values. The model under-predicted the KM cumulative incidence of T2DM in the first eight years (simulated to observed ratio less than one), predicted reasonably well from years eight through 11, and over-predicted from years 12 through 15 (simulated to observed ratio greater than one) for both the placebo and intervention arm. As described in the manuscript discussion, although the external validation indicates some discrepancies between the model

predictions and the external dataset, interpretation of these discrepancies is difficult. External validation against other independent datasets that use HbA1c to diagnose T2DM would provide additional insights and will be an important future step in determining the model's predictive performance.

2.2 Case study results: Cost-effectiveness of a potential diabetes prevention program

Figure 5 shows the distribution of the PSA results on the cost-effectiveness plane with a WTP threshold of $30,000[58] represented by the solid gray line. The PSA results indicate that the intervention is highly likely to be cost-effective at a WTP threshold of $30,000.

Figure 5: Cost-effectiveness plane

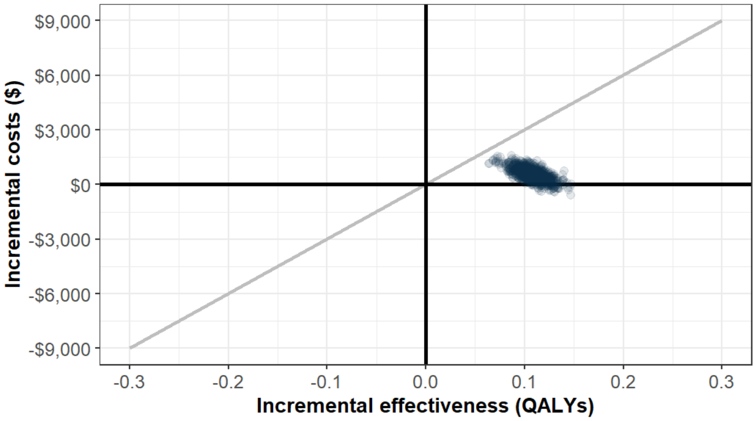

Notes: 1,000 iterations of 5,100 patients per iteration; QALY: quality-adjusted life-year

Figure 6 below shows the expected INMB and 95% UI for the intervention in one-year cycles up to 15 years. For each yearly cycle, the NMB is calculated using the average cost and QALYs over all prior years up to and including the current annual cycle. For each year, the most cost-effective and hence the favoured strategy is that which yields the greatest NMB; therefore, when the INMB for the intervention becomes positive, the intervention is the favoured strategy. The point of intersection of the INMB line at zero on the y-axis indicates the minimum time frame required for the intervention to be considered cost-effective. As shown in Figure 6, the intervention becomes cost-effective in year ten.

FIGURE 6: Incremental net monetary benefit of the intervention vs standard care by year

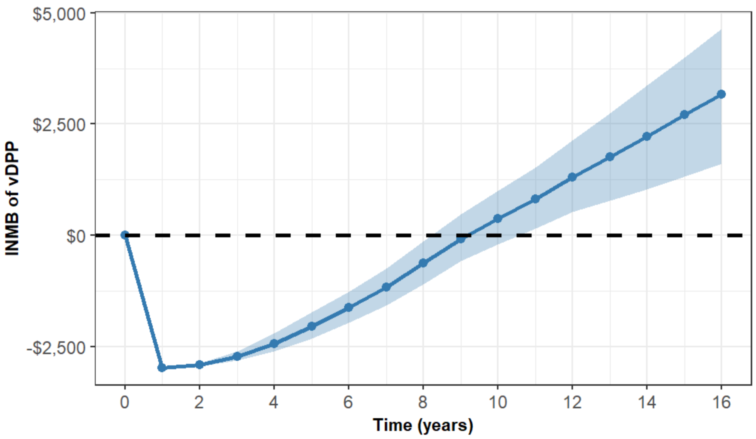

Figure 7 indicates the cumulative T2DM event rate over the 15-year simulation. The cumulative event rate is calculated as the cumulative number of incident T2DM cases divided by the cumulative person-years (PYs) at risk of T2DM (i.e., alive and do not have T2DM).

FIGURE 7: Cumulative T2DM rate per 100,000 person-years

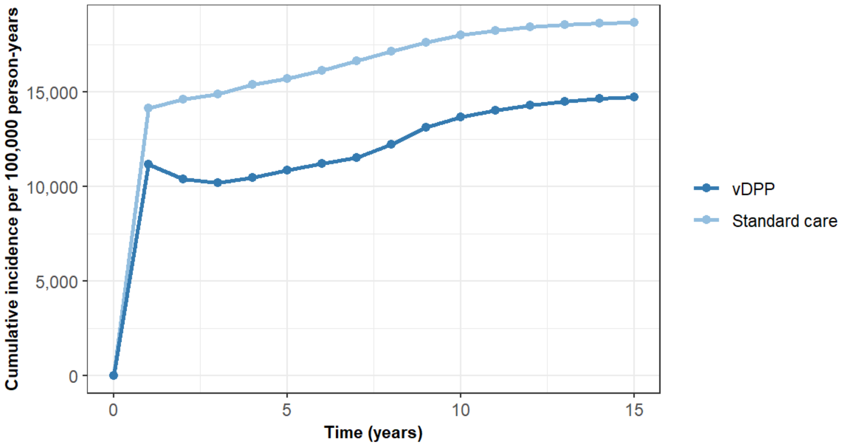

Figure 8 shows how the range of the NMB values from the PSA are distributed for each strategy. The distribution for the intervention is shifted to the right as compared to standard care, indicating that, on average, the NMB for the intervention is higher than for standard care. The NMB distribution for the standard care has a slightly higher peak value, which reflects greater certainty in the expected NMB.

FIGURE 8: Net monetary benefit density plot

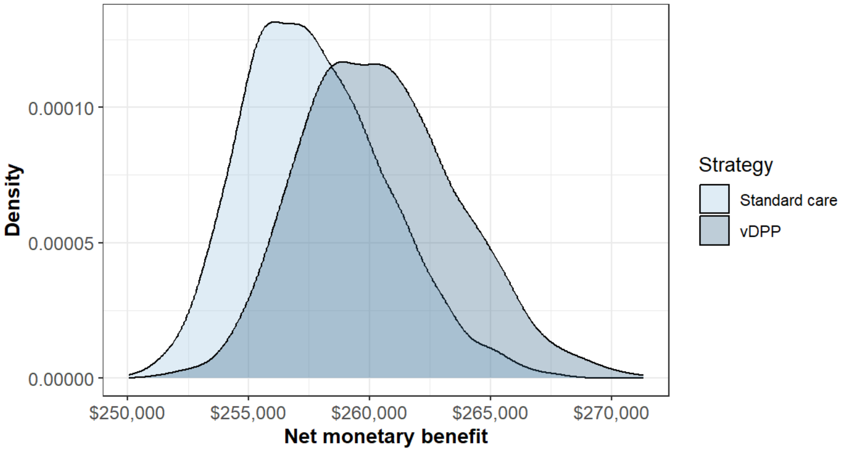

Figure 9 shows the EVPI over a range of WTP values. The EVPI at a WTP of $30,000 per QALY is $0 per patient. At a WTP of $5,000 per QALY, approximately the threshold where the intervention becomes cost-effective, the EVPI reaches its maximum value of $148 per patient.

FIGURE 9: Expected value of perfect information

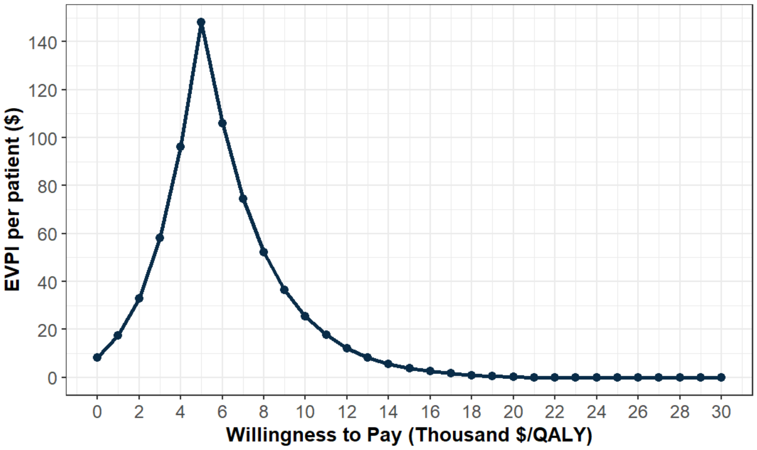

Comparisons to other studies

Several other studies examined the cost-effectiveness of lifestyle interventions to prevent or delay the onset of T2DM, with results that can be compared to our findings. Herman et al. (2005)[6] examined the cost-effectiveness of the DPP lifestyle intervention over a lifetime time horizon in the US. They used a cohort Markov model that simulated progression from pre-T2DM to T2DM and complications once patients transitioned to T2DM. As in our study, they found the lifestyle intervention was highly cost-effective, with a cost per QALY of $1,100. In another

study, Caro et al. (2004)[59] evaluated an intensive lifestyle intervention program in Canada over a 10-year time horizon using a simple three-state Markov model. They also found the intervention was cost-effective, with increases in both costs and life-years resulting in an incremental cost-effectiveness ratio (ICER) of $749. Lindgren et al. (2007)[5] used a simulation model to assess an intensive lifestyle modification program in Sweden. They found the program was cost-saving over a lifetime horizon with an ICER of €2,363. These differences can likely be attributed to differences in the baseline population and/or differences in program and health system costs in Sweden compared to the US and Canada.

Our findings were consistent with the results of comparable studies, showing that the intervention was cost-effective. However, comparable studies used models of varying complexity, and few of these models simulated correlated progression of multiple T2DM risk factors over time and simulated comorbidities both before and after patients transitioned to T2DM, as our model does.

Completed CHEERS 2022 checklist

Topic	No.	Item	Location where item is reported
**Title**			
	1	Identify the study as an economic evaluation and specify the interventions being compared.	page 1 *note: this manuscript does not present a comprehensive cost-effectiveness analysis, but a case study to demonstrate the model's potential applications
**Abstract**			
	2	Provide a structured summary that highlights context, key methods, results, and alternative analyses.	page 2
**Introduction**			
**Background and objectives**	3	Give the context for the study, the study question, and its practical relevance for decision making in policy or practice.	page 4, paragraph 2
**Methods**			

Topic	No.	Item	Location where item is reported
**Health economic analysis plan**	4	Indicate whether a health economic analysis plan was developed and where available.	NA *note: this manuscript does not present a comprehensive cost-effectiveness analysis, but a case study to demonstrate the model's potential applications
**Study population**	5	Describe characteristics of the study population (such as age range, demographics, socioeconomic, or clinical characteristics).	Main text page 6, paragraph 1; supplemental material pages 4-6
**Setting and location**	6	Provide relevant contextual information that may influence findings.	Main text page 12, paragraph 2; supplemental material page 32, paragraph 3
**Comparators**	7	Describe the interventions or strategies being compared and why chosen.	Main text page 12, paragraph 3
**Perspective**	8	State the perspective(s) adopted by the study and why chosen.	Supplemental material page 32, paragraph 4
**Time horizon**	9	State the time horizon for the study and why appropriate.	Main text page 12, paragraph 2; supplemental material page 32, paragraph 3
**Discount rate**	10	Report the discount rate(s) and reason chosen.	Main text page 12, paragraph 4
**Selection of outcomes**	11	Describe what outcomes were used as the measure(s) of benefit(s) and harm(s).	Main text page 8-9; supplemental material page 11
**Measurement of outcomes**	12	Describe how outcomes used to capture benefit(s) and harm(s) were measured.	Main text page 8-9; supplemental material page 11
**Valuation of outcomes**	13	Describe the population and methods used to measure and value outcomes.	Main text page 8-9; supplemental material page 11
**Measurement and valuation of resources and costs**	14	Describe how costs were valued.	Main text page 8; supplemental material pages 9 - 10
**Currency, price date, and conversion**	15	Report the dates of the estimated resource quantities and unit costs, plus the currency and year of conversion.	Main text page 3

Topic	No.	Item	Location where item is reported
**Rationale and description of model**	16	If modelling is used, describe in detail and why used. Report if the model is publicly available and where it can be accessed.	Main text pages 4-5
**Analytics and assumptions**	17	Describe any methods for analysing or statistically transforming data, any extrapolation methods, and approaches for validating any model used.	Main text pages 9-11
**Characterising heterogeneity**	18	Describe any methods used for estimating how the results of the study vary for subgroups.	NA
**Characterising distributional effects**	19	Describe how impacts are distributed across different individuals or adjustments made to reflect priority populations.	Main text pages 6-7
**Characterising uncertainty**	20	Describe methods to characterise any sources of uncertainty in the analysis.	Main text page 9
**Approach to engagement with patients and others affected by the study**	21	Describe any approaches to engage patients or service recipients, the general public, communities, or stakeholders (such as clinicians or payers) in the design of the study.	NA
**Results**			
**Study parameters**	22	Report all analytic inputs (such as values, ranges, references) including uncertainty or distributional assumptions.	supplemental material pages 34 - 44
**Summary of main results**	23	Report the mean values for the main categories of costs and outcomes of interest and summarise them in the most appropriate overall measure.	Main text page 14
**Effect of uncertainty**	24	Describe how uncertainty about analytic judgments, inputs, or projections affect findings. Report the effect of choice of discount rate and time horizon, if applicable.	supplemental material pages 50 - 53

Topic	No.	Item	Location where item is reported
**Effect of engagement with patients and others affected by the study**	25	Report on any difference patient/service recipient, general public, community, or stakeholder involvement made to the approach or findings of the study	NA
**Discussion**			
**Study findings, limitations, generalisability, and current knowledge**	26	Report key findings, limitations, ethical or equity considerations not captured, and how these could affect patients, policy, or practice.	Main text pages 15 - 18
**Other relevant information**			
**Source of funding**	27	Describe how the study was funded and any role of the funder in the identification, design, conduct, and reporting of the analysis	Main text page 19
**Conflicts of interest**	28	Report authors conflicts of interest according to journal or International Committee of Medical Journal Editors requirements.	Main text page 19

From: Husereau D, Drummond M, Augustovski F, et al. Consolidated Health Economic Evaluation Reporting Standards 2022 (CHEERS 2022) Explanation and Elaboration: A Report of the ISPOR CHEERS II Good Practices Task Force. Value Health 2022;25. doi:10.1016/j.jval.2021.10.008

References

1. American Diabetes Association Consensus Panel. Guidelines for computer modeling of diabetes and its complications. *Diabetes care* 2004; 27: 2262-2265. DOI: 10.2337/diacare.27.9.2262.

2. Hoerger TJ, Hilscher R, Neuwahl S, et al. A New Type 2 Diabetes Microsimulation Model to Estimate Long-Term Health Outcomes, Costs, and Cost-Effectiveness. *Value Health* 2023 2023/05/27. DOI: 10.1016/j.jval.2023.05.013.

3. Hayes AJ, Leal J, Gray AM, et al. UKPDS Outcomes Model 2: a new version of a model to simulate lifetime health outcomes of patients with type 2 diabetes mellitus using data from the 30 year United Kingdom Prospective Diabetes Study: UKPDS 82. *Diabetologia* 2013; 56: 1925-1933. DOI: 10.1007/s00125-013-2940-y.

4. Dall TM, Storm MV, Semilla AP, et al. Value of lifestyle intervention to prevent diabetes and sequelae. *Am J Prev Med* 2014; 48: 271-280. DOI: 10.1016/j.amepre.2014.10.003.

5. Lindgren P, Lindström J, Tuomilehto J, et al. Lifestyle intervention to prevent diabetes in men and women with impaired glucose tolerance is cost-effective. *Int J Technol Assess Health Care* 2007; 23: 177-183. DOI: 10.1017/s0266462307070286.

6. Herman WH, Hoerger TJ, Brändle M, et al. The Cost-Effectiveness of Lifestyle Modification or Metformin in Preventing Type 2 Diabetes in Adults with Impaired Glucose Tolerance. *Ann Intern Med medicine* 2005; 142: 323-332. DOI: 10.7326/0003-4819-142-5-200503010-00007.

7. Mount Hood Diabetes Challenge Network. Economics, simulation modelling & diabetes, https://www.mthooddiabeteschallenge.com/ (2022, accessed 2022 Jun 1).

8. Breeze PR, Thomas C, Squires H, et al. The impact of Type 2 diabetes prevention programmes based on risk-identification and lifestyle intervention intensity strategies: a cost-effectiveness analysis. *Diabet Med* 2017; 34: 632-640. DOI: 10.1111/dme.13314.

9. Diabetes Prevention Program Research Group, Knowler WC, Fowler SE, et al. 10-year follow-up of diabetes incidence and weight loss in the Diabetes Prevention Program Outcomes Study. *Lancet* 2009; 374: 1677-1686. 2009/10/29. DOI: 10.1016/S0140-6736(09)61457-4.

10. Breeze P, Squires H, Chilcott J, et al. A statistical model to describe longitudinal and correlated metabolic risk factors: the Whitehall II prospective study. *J Public Health (Oxf)* 2015; 38: 679-687. DOI: 10.1093/pubmed/fdv160.

11. Leal J, Alva ML, Gregory V, et al. Estimating risk factor progression equations for the UKPDS Outcomes Model 2 (UKPDS 90). *Diabet Med* 2021; 38: e14656. DOI: 10.1111/dme.14656.

12. The Diabetes Prevention Program. Design and methods for a clinical trial in the prevention of type 2 diabetes. *Diabetes Care* 1999; 22: 623-634. 1999/04/06. DOI: 10.2337/diacare.22.4.623.

13. Table 98-10-0351-01 Visible minority by gender and age: Canada, provinces and territories. In: Statistics Canada, (ed.). Ottawa (ON)2023.

14. Government of Alberta (GoA). *2021 Census of Canada - Indigenous People*. Report no. 978-1-4601-5664-3, 2023. Edmonton (AB): GoA.

15. Crowshoe L, Dannenbaum D, Green M, et al. Type 2 Diabetes and Indigenous Peoples. *Can J Diabe* 2018; 42: S296-S306. DOI: 10.1016/j.jcjd.2017.10.022.

16. Government of Canada. *Canadian Tobacco and Nicotine Survey (CTNS): summary of results for 2020*. 2022. Ottawa (ON): Government of Canada.

17. National Kidney Foundation Inc. Estimated Glomerular Filtration Rate (eGFR), https://www.kidney.org/atoz/content/gfr (2023, accessed 2023 Jul 17).

18. Canadian Blood Services. What you need to know about hemoglobin, anemia, iron and hemochromatosis, https://www.blood.ca/en/blood/am-i-eligible-donate-blood/abcs-eligibility/what-you-need-to-know-about-hemoglobin-hemochromatosis-iron-anemia (2023, accessed 2023 Jul 17).

19. American Heart Association Inc. What Your Cholesterol Levels Mean, https://www.heart.org/en/health-topics/cholesterol/about-cholesterol/what-your-cholesterol-levels-mean (2023, accessed 2023 Jul 17).

20. American Heart Association Inc. Target Heart Rates Chart, https://www.heart.org/en/healthy-living/fitness/fitness-basics/target-heart-rates (2023, accessed 2023 Jul 17).

21. American Heart Association Inc. Understanding Blood Pressure Readings, https://www.heart.org/en/health-topics/high-blood-pressure/understanding-blood-pressure-readings (2023, 2023 Jul 17).

22. Canadian Cancer Society. Low white blood cell count (neutropenia), https://cancer.ca/en/treatments/side-effects/low-white-blood-cell-count (2023, 2023 Jul 17).

23. de Simone G, Devereux RB, Roman MJ, et al. Relation of obesity and gender to left ventricular hypertrophy in normotensive and hypertensive adults. *Hypertension* 1994; 23: 600-606. DOI: 10.1161/01.hyp.23.5.600.

24. Table 3: Hypertension prevalence, awareness, treatment and control, by sex and age, household population aged 20 to 79 years, Canada, combined 2012 to 2015. In: Statistics Canada, (ed.). Ottawa (ON)2019.

25. Public Health Agency of Canada (PHAC). *Heart Disease in Canada* 2022. Ottawa (ON): PHAC.

26. Public Health Agency of Canada (PHAC). *Stoke in Canada: Highlights from the Canadian Chronic Disease Surveillance System*. Report no. 978-0-660-08889-1, 2019. Ottawa (ON): PHAC.

27. Public Health Agency of Canada (PHAC). *Heart disease in Canada: Highlights from the Canadian Chronic Disease Surveillance System*. 2017. Ottawa (ON).

28. Consumer price index (CPI). In: Statistics Canada, (ed.). Ottawa (ON)2023.

29. Canadian Agency for Drugs and Technologies in Health (CADTH). *Guidelines for the economic evaluation of health technologies: Canada. 4th ed*. 2017. Ottawa (ON): CADTH.

30. O'Reilly D, Hopkins R, Blackhouse G, et al. *Development of an Ontario Diabetes Economic Model (ODEM) and Application to a Multidisciplinary Primary Care Diabetes Management Program*. 2006. Hamilton (ON): Ontario Ministry of Health and Long-term Care.

31. O'Brien JA, Patrick AR and Caro JJ. Cost of managing complications resulting from type 2 diabetes mellitus in Canada. *BMC Health Serv Res* 2003; 3: 7. 2003/03/28. DOI: 10.1186/1472-6963-3-7.

32. Choi J, Booth G, Jung HY, et al. Association of diabetes with frequency and cost of hospital admissions: a retrospective cohort study. *CMAJ open* 2021; 9: E406-E412. DOI: 10.9778/cmajo.20190213.

33. Rosella LC, Lebenbaum M, Fitzpatrick T, et al. Impact of diabetes on healthcare costs in a population-based cohort: a cost analysis. *Diabet Med* 2016; 33: 395-403. DOI: 10.1111/dme.12858.

34. University of Alberta. School of Public Health. Alberta PROMS and EQ-5D Research Support Unit. *Alberta Population Norms for EQ-5D-5L*. 2018. Edmonton (AB): University of Alberta.

35. Sullivan PW, Lawrence WF and Ghushchyan V. A national catalog of preference-based scores for chronic conditions in the United States. *Medical care* 2005: 736-749. DOI: 10.1097/01.mlr.0000172050.67085.4f.

36. Alva M, Gray A, Mihaylova B, et al. The effect of diabetes complications on health-related quality of life: the importance of longitudinal data to address patient heterogeneity. *Health Econ* 2014; 23: 487-500. DOI: 10.1002/hec.2930.

37. O'Reilly DJ, Xie F, Pullenayegum E, et al. Estimation of the impact of diabetes-related complications on health utilities for patients with type 2 diabetes in Ontario, Canada. *Qual Life Res* 2011; 20: 939-943. DOI: 10.1007/s11136-010-9828-9.

38. Diabetes Canada. *Diabetes Canada 2018 clinical practice guidelines for the prevention and management of diabetes in Canada*. Diabetes Canada, 2018.

39. Heianza Y, Arase Y, Fujihara K, et al. Longitudinal trajectories of HbA1c and fasting plasma glucose levels during the development of type 2 diabetes: the Toranomon Hospital Health Management Center Study 7 (TOPICS 7). *Diabetes Care* 2012; 35: 1050-1052. DOI: 10.2337/dc11-1793.

40. Heianza Y, Arase Y, Fujihara K, et al. Screening for pre-diabetes to predict future diabetes using various cut-off points for HbA(1c) and impaired fasting glucose: the Toranomon Hospital Health Management Center Study 4 (TOPICS 4). *Diabet Med* 2012; 29: e279-285. 2012/04/19. DOI: 10.1111/j.1464-5491.2012.03686.x.

41. Neumann A, Schwarz P and Lindholm L. Estimating the cost-effectiveness of lifestyle intervention programmes to prevent diabetes based on an example from Germany: Markov modelling. *Cost Eff Resour Alloc.* 2011; 9: 17. DOI: 10.1186/1478-7547-9-17.

42. Anderson KM, Odell PM, Wilson PW, et al. Cardiovascular disease risk profiles. *Am Heart J* 1991; 121: 293-298. DOI: 10.1016/0002-8703(91)90861-b.

43. D'Agostino Sr RB, Vasan RS, Pencina MJ, et al. General cardiovascular risk profile for use in primary care: the Framingham Heart Study. *Circulation* 2008; 117: 743-753. DOI: 10.1161/CIRCULATIONAHA.107.699579.

44. Dillingham TR, Pezzin LE and Shore AD. Reamputation, mortality, and health care costs among persons with dysvascular lower-limb amputations. *Arch Phys Med Rehabil* 2005; 86: 480-486. 2005/03/11. DOI: 10.1016/j.apmr.2004.06.072.

45. Hippisley-Cox J and Coupland C. Predicting the risk of Chronic Kidney Disease in Men and Women in England and Wales: prospective derivation and external validation of the QKidney® Scores. *BMC Fam Pract* 2010; 11: 1-13.

46. Vemmos KN, Bots M, Tsibouris P, et al. Prognosis of stroke in the south of Greece: 1 year mortality, functional outcome and its determinants: the Arcadia Stroke Registry. *J Neurol Neurosurg Psychiatry* 2000; 69: 595-600. DOI: 10.1136/jnnp.69.5.595.

47. AMI statistics. Based on county of residence, Case fatality of AMI. Deaths within 365 days (%), Entire Sweden, Age interval: 20-85+. In: Welfare NBoHa, (ed.). Stockhom (Sweden)2022.

48. McAlister FA, Bakal JA, Kaul P, et al. Changes in Heart Failure Outcomes After a Province-Wide Change in Health Service Provision A Natural Experiment in Alberta, Canada. *Circ Heart Fail* 2013; 6: 76-82. DOI: doi:10.1161/CIRCHEARTFAILURE.112.971119.

49. Table 13-10-0392-01 Deaths and age-specific mortality rates by selected grouped causes. In: Statistics Canada, (ed.). Ottawa (ON)2022.

50. DeGuire J, Clarke J, Rouleau K, et al. *Blood pressure and hypertension*. 2019. Ottawa (ON): Statistics Canada.

51. Venditti EM, Bray GA, Carrion-Petersen ML, et al. First versus repeat treatment with a lifestyle intervention program: attendance and weight loss outcomes. *Int J Obes (Lond)* 2008; 32: 1537-1544. DOI: 10.1038/ijo.2008.134.

52. Punthakee Z, Goldenberg R and Katz P. Definition, Classification and Diagnosis of Diabetes, Prediabetes and Metabolic Syndrome. *Can J Diabetes* 2018; 42 Suppl 1: S10-s15. 2018/04/14. DOI: 10.1016/j.jcjd.2017.10.003.

53. Centers for Disease Control and Prevention (CDC). *Centers for Disease Control and Prevention Diabetes Prevention Recognition Program: Standard and Operating Procedures*. 2021. CDC.

54. Ely EK, Gruss SM, Luman ET, et al. A National Effort to Prevent Type 2 Diabetes: Participant-Level Evaluation of CDC's National Diabetes Prevention Program. *Diabetes Care* 2017; 40: 1331-1341. 2017/05/14. DOI: 10.2337/dc16-2099.

55. Cannon MJ, Masalovich S, Ng BP, et al. Retention Among Participants in the National Diabetes Prevention Program Lifestyle Change Program, 2012-2017. *Diabetes Care* 2020; 43: 2042-2049. 2020/07/04. DOI: 10.2337/dc19-2366.

56. Ritchie ND, Baucom KJW and Sauder KA. Current Perspectives on the Impact of the National Diabetes Prevention Program: Building on Successes and Overcoming Challenges. *Diabetes Metab Syndr Obes* 2020; 13: 2949-2957. DOI: 10.2147/DMSO.S218334.

57. Healthwise Staff. Prediabetes, https://myhealth.alberta.ca/Health/Pages/conditions.aspx?hwid=uz1410&lang=en-ca#:~:text=Your%20treatment%20for%20prediabetes%20will,healthy%20foods%2C%20and%20getting%20active. (2023, accessed 2023 Dec 12).

58. Ochalek J, Lomas J and Claxton K. *Assessing health opportunity costs for the Canadian health care systems*. 2018.

59. Caro JJ, Getsios D, Caro I, et al. Economic evaluation of therapeutic interventions to prevent Type 2 diabetes in Canada. *Diabet Med.* 2004; 21: 1229-1236. DOI: 10.1111/j.1464-5491.2004.01330.x.
